# Pan-cancer analysis of a novel indicator of necroptosis with its application in human cancer

**DOI:** 10.18632/aging.204307

**Published:** 2022-09-26

**Authors:** Aibin Liu, Yanyan Li, Lin Shen, Na Li, Liangfang Shen, Zhanzhan Li

**Affiliations:** 1 Department of Geriatrics, Xiangya Hospital, Central South University, Changsha 410008, Hunan Province, P.R. China; 2 Department of Nursing, Xiangya Hospital, Central South University, Changsha 410008, Hunan Province, P.R. China; 3 Department of Oncology, Xiangya Hospital, Central South University, Changsha 410008, Hunan Province, P.R. China; 4 National Clinical Research Center for Geriatric Disorders, Xiangya Hospital, Central South University, Changsha 410008, Hunan Province, P.R. China

**Keywords:** necroptosis, pan-cancer, immunity, TCGA, bioinformatics

## Abstract

As a type of programmed cell death, necroptosis is thought to play a dual role in tumorigenesis. However, a comprehensive assessment of necroptosis-related regulators across human cancers has not been reported. Therefore, in this study, we established a quantitative index to evaluate the necroptosis rate and determine its correlations with clinical prognosis, signaling pathways and molecular features, immune cell infiltration and regulation, immunotherapy, and chemotherapy sensitivity across cancers. Our results indicated that the necroptosis score can act as a favorable or risky prognostic factor in various cancer types. A gene set variation analysis suggested that necroptosis is significantly associated with immune- and inflammation-related signaling pathways, cell growth and apoptosis, and energy metabolism. Furthermore, necroptosis can affect the tumor microenvironment and immunity regulation, and the effect of necroptosis on immunity is different in different tumor types. There is crosstalk between components of necroptosis, pyroptosis, ferroptosis and autophagy pathways in multiple types of cancers. Finally, the necroptosis rate can be an indicator of immunotherapy effectiveness in multiple cancers and can affect the chemotherapy sensitivity of cancer cells. Our study presents a characterization of necroptosis across human cancers, highlights the potential necroptotic effects on immune regulation, and provides new insights into the development of individualized tumor treatments and clinical applications of immunotherapy.

## INTRODUCTION

Traditionally, apoptosis was the sole regulated cell death pathway [1]. Furthermore, a corresponding cell death pathway, necrosis, was considered a form of unprogrammed or “dumb” cell death, which ultimately led to loss of membrane integrity and passive release of cellular contents [2]. It is now clear that there is a nonapoptotic form of cell death that has evolved to detect pathogens and promote tissue repair; this regulated cell death is known as necroptosis [3]. Necroptosis, which is neither necrosis nor apoptosis, is an alternative mode of programmed cell death that mimics characteristics of both apoptosis and necrosis. Dysregulation of necroptosis is a key factor in many inflammatory diseases [4, 5]. Recent studies have revealed an important role played by necroptosis in tumorigenesis and metastasis, suggesting the potential of targeting necroptosis as a new tumor therapy [6]. In the body, certain cells die silently in an orderly fashion by activating apoptosis, which is a cell suicide program. Other cells, often when infected by a virus, undergo a messier and more violent form of death, necroptosis, in which the immune system attacks and kills the body's own cells [7, 8]. In recent years, studies have shown that activating necroptosis in cancer cells induces a similar immune system attack on tumors, which indicates that necroptosis, a type of programmed cell death, is involved in the body's immune regulation [9, 10]. Although the molecular mechanisms of necroptosis have been extensively studied, the details of necroptosis regulation and function in tumorigenesis and metastasis are not fully understood.

Therefore, in the current study, we constructed a novel indicator to describe the status of necroptosis based on necroptosis-related gene expression profiling. Using this indicator, we analyzed the relationship between necroptosis and prognosis, signaling pathways and molecular features, immune cell infiltration and regulation, immunotherapy, and chemotherapy sensitivity in different tumors. We thus comprehensively evaluated the biological function and clinical relevance of necroptosis across cancers. Our results reveal the functional and mechanistic landscape of necroptosis in cancer and provide new insights for the precise treatment of tumors in the future.

## MATERIALS AND METHODS

### Data sources

**Figure 1:**
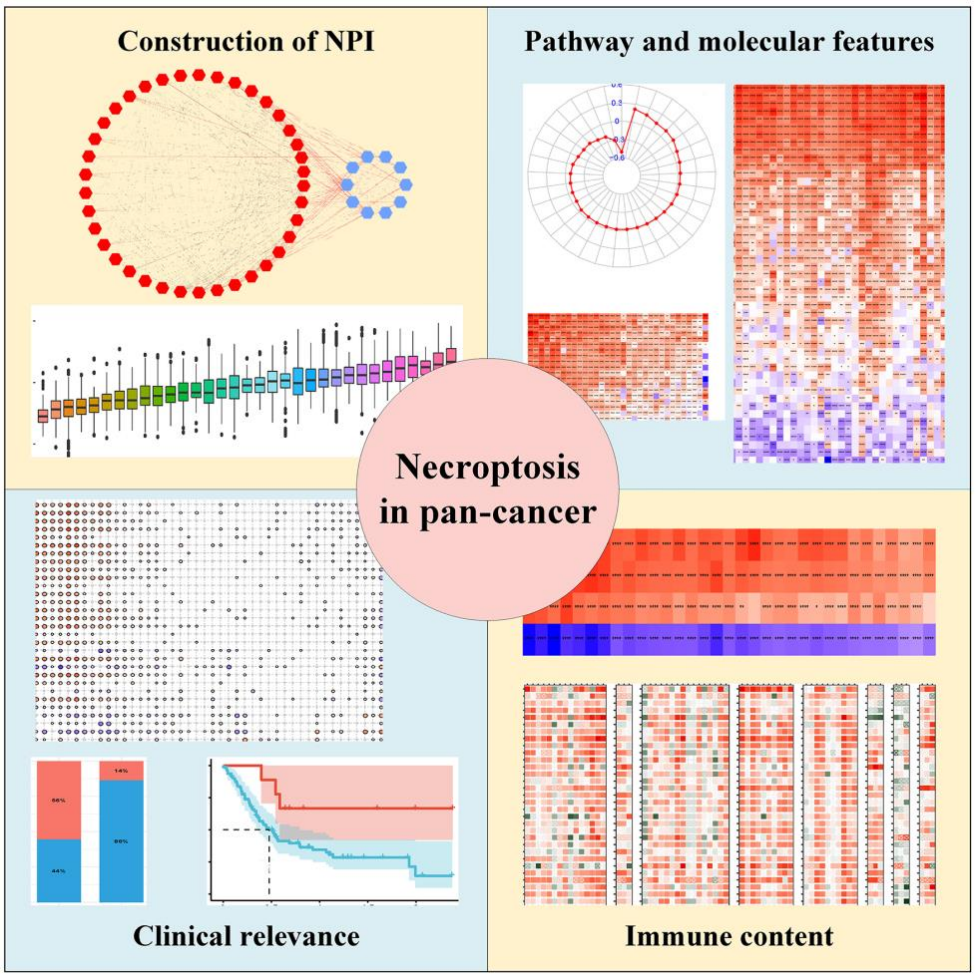
The overall overview of the study.

We downloaded the transcriptional data of 10496 samples from the UCSC Xena database (https://xenabrowser.net/datapages/) and normal expression data from GTEX project database. The list of cancer types is presented in Supplementary Table 1. The clinical parameters and follow-up information was also obtained. The gene-level copy number, DNA methylation, and somatic mutation data were also downloaded. The tumor mutation burden was calculated according to the single-nucleotide variation profiles of the cancers. Microsatellite instability data were obtained from previously published data. Necroptosis regulator data were obtained from previous studies and the Molecular Signatures Database. We ultimately included 67 necroptosis regulators from previous study [11]. The list of genes is provided in Additional file 1: Supplementary Table 2. Figure 1 presents the overall overview of our study.

### Establishment of the necroptosis score (NPS) in cancers

Using the necroptosis regulator genes (NRGs), we first constructed a protein–protein interaction (PPI) network using the STRING database (STRING: functional protein association networks (string-db.org)), and we identified the hub genes by inputting the PPI data into Cytoscape software. Next, we explored the correlations among the necroptosis regulators in all cancers. Analyses of differentially expressed NRGs were also performed to compare the expression differences in tumor and normal samples (GTEX data). Then, we explored the function and pathway enrichment of these NRGs by performing GO and KEGG analyses. Subsequently, we evaluated the single-nucleotide variant (SNV), copy number variation (CNV), and methylation status in cancers. We also identified the prognostic roles played by these NRGs in cancers by log-rank test. Finally, we performed single-sample gene set variation analysis (ssGSEA) using the NRG set, and we assigned a necroptosis score (NPS) to each sample according to the enrichment levels. We completed the calculation using the R “GSEABase” packages. In ssGSEA, gene expression values for a given sample are rank-normalized, and an enrichment score is produced using the Empirical Cumulative Distribution Functions (ECDF) of the genes in the signature and the remaining genes. For a given signature G of size NG and single sample S, of the data set of N genes, the genes are replaced by their ranks according their absolute expression from high to low: An enrichment score ES (G, S) is obtained by a sum (integration) of the difference between a weighted ECDF of the genes in the signature and the ECDF of the remaining genes PNG [12]:


$$ES(G,S)=\sum_{i=1}^{N}\left[P_{G}^{w}(G,S,\;i)-P_{N_{G}}(G,S,i)\right]$$

### Expression profile based on the NPS and its correlation with clinical prognosis in cancers

After obtaining the NPS of each sample, we first evaluated the expression level in 33 cancers and the differential NPSs between tumor samples and normal samples in different cancers. Wilcoxon tests were performed to compare tumor and normal tissues in each cancer. The tumor samples were categorized into a high-NPS group and a low-NPS group on the basis of the optimal cutoff NPS value for each cancer. We evaluated the associations between NPS and prognosis in each cancer. The four types of prognosis were overall survival (OS), disease-specific survival (DSS), disease-progression interval (DFI), and progression-free interval (PFI). A Kaplan–Meier curve was assessed to compare survival curves, and univariate Cox regression was performed to calculate the hazard ratio (HR) and the corresponding confidence interval (CI). *P*<0.05 was considered to be significant.

### Associations between NPS and related pathways and molecular features in cancers

We also performed gene set variation analysis (GSVA) using R packages (GSVA) and the latest HALLMARK pathways dataset from the MsigDB, including fifty common signaling pathways. As necroptosis is a type of programmed cell death, we explored the correlation between the NPS and certain cell death-related gene sets, including genes involved in pyroptosis, autophagy, and ferroptosis. The results were visualized in a heatmap.

### The NPS and immune status in cancers

To explore the association between the NPS and immune status in each cancer, we followed three different methods. First, we used the ESTIMATE algorithm, which depicts a tumor microenvironment based on four parameters: immune, stromal, ESTIMATE scores, and tumor purity. The second method involved evaluating the correlations between NPS and the TME signature [13]. The third method was based on the CIBERSORT algorithm, which allows for the sensitive and specific discrimination of 22 human immune cell phenotypes. Finally, we explored the associations between the NPS and immune regulation genes, including MHC genes, immune-activated and inhibited genes, immune checkpoint genes, chemokines, and chemokine receptor genes, using Pearson correlation.

### Effect of the NPS on immunotherapy

We performed a ssGSEA for the following datasets: GSE32894 (urothelial carcinoma), GSE135222 (advanced non-small-cell lung carcinoma patients), GSE176307 (metastatic urothelial cancer), and the Supplementary Materials of previous studies for KIRC (PMID: 32472114) [14]. Patients were categorized into a high-NPS group and a low-NPS group on the basis of the optimal cutoff value (minimum P value). Then, we performed Kaplan–Meier analysis to evaluate the effect of the NPS on prognosis in patients who received immunotherapy.

### The NPS and chemotherapy sensitivity in cancers

Using the GDSC and CTRP databases, we performed Pearson correlation analysis to obtain the correlations between mRNA expression of NPGs and drug IC50. The P value was adjusted on the basis of the false discovery rate (FDR). The correlations were visualized with a bubble plot. Negative correlations indicated chemotherapy sensitivity, and positive correlations indicated chemotherapy resistance.

### Data availability

All data can be download from UCSC Xena database (https://portal.gdc.cancer.gov/).

## RESULTS

### Landscape of necroptosis regulators across cancers

**Figure 2:**
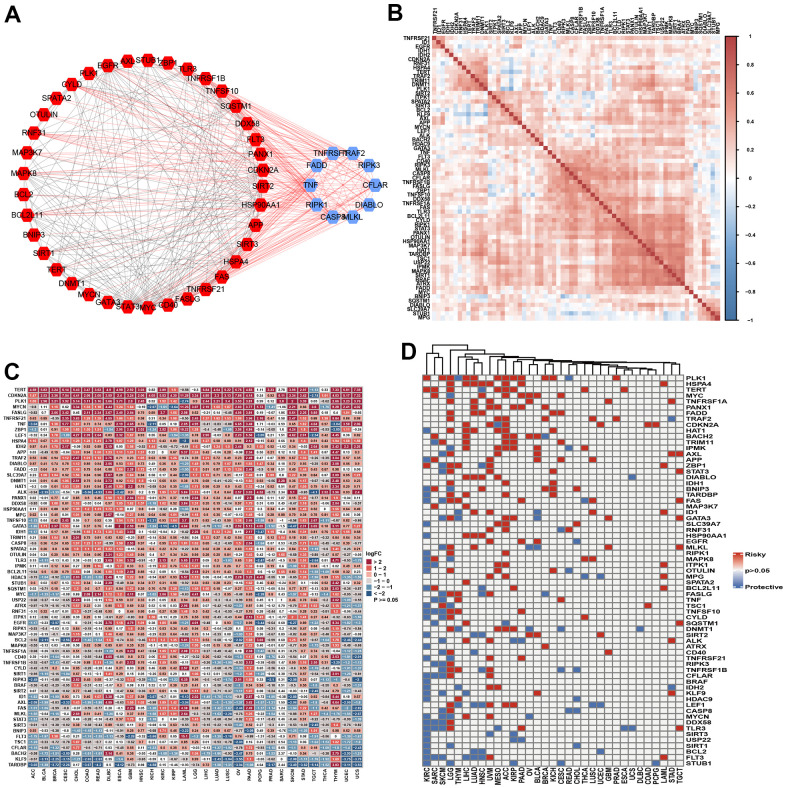
**The landscape of necroptosis regulators.** (**A**) Protein-protein interaction among necroptosis regulators (Blue: Top 10 hub genes; Red: non-hub genes). (**B**) Heatmap showed the expression profiling of necroptosis regulators between tumors and normal samples. (**C**) Heatmap indicated the correlations among necroptosis regulators in pan-cancer. (**D**) The prognosis role of necroptosis regulators in pan-cancer using log-rank method.

We ultimately obtained 67 necroptosis regulator genes. Figure 2A presents the PPI associated with these NRGs. We identified the 10 most interactive hub genes using maximal clique centrality methods: TNFRSF1A, RAF2, RIPK3, CFLAR, DIABLO, MLKL, CASP8, RIPK1, TNF, and FADD. A correlation heatmap of these genes is presented in Figure 2B, and it indicates that more positive associations were found among these NRGs. STUB1, KLF9, and BNPI3 tended to be negatively associated with other genes. The GO enrichment analysis indicated that these NRGs were mainly enriched in necroptotic progression, the extrinsic apoptotic signaling pathway, DNA-binding transcription factor activity, tumor necrosis factor receptor superfamily binding, and ubiquitin protein ligase binding and activity (Supplementary Figure 1A). The KEGG analysis indicated that these genes were involved in the TNF signaling pathway, RIG-I-like receptor, NF-kappa B, NOD-like receptor, FoxO, Toll-like receptor, IL-17, MAPK, and adipocytokine signaling pathways (Supplementary Figure 1B).

The single-nucleotide variation (SNV) analysis indicated that the variant classification was mainly missense, nonsense, frame shift, splice, and frame shift insertions. Most SNV classes included a C>T mutation. The 10 most mutated genes were ATRX BRAF, IDH1, EGFR, CDKN2A, ALK, HADC9, CASP8, GATA3, and FLT3 (Supplementary Figure 2A). BRAF showed higher mutation frequencies in SKCM and THCA. The mutation frequencies of ATRX and IDH1 were the highest in LGG. EGFR mutations were mainly found in GBM, UCEC, SKCM, LGG, LUAD, and STAD. The 10 most mutated genes were found in similar cancer types (Supplementary Figure 2B, 2C). The following genes showed the highest copy number variations: MTC, ID1, ZBP1, SPATA2, CD40, EGFR, HDAC9, FASLG, BRAF, TRIM11, TERT, TNFSF10, OTULIN, and TRNFRSF1A. These genes showed a high CNV percentage in each cancer (Supplementary Figure 3A). The Spearman correlation analysis indicated that CNV was correlated with mRNA expression (Supplementary Figure 3B). We then explored the differential expression levels of the NGRs between tumor and normal samples. TERT, CDKN2A, PLK1, FASLG, TNFRSF21, TNF, ZBP1, LEF1, HSPA4, and IDH2 were highly expressed in most tumor tissues, while TARDBP, KLF9, BACH2, CFLAR, TSC1, FLT3, and AXL were expressed at low levels in most cancers (Figure 2C). We further explored the correlation of these NGRs with clinical outcomes by performing log-rank tests. NGRs played protective roles in KIRC, SARC and SKCM and risk-enhancing roles in LGG, THYM, LIHC, LUAD, UVM, and ACC. NGRs did not seem to be associated with the prognosis of certain cancers, such as COAD, DLBC, UCS, and ESCA (Figure 2D). Finally, we evaluated the methylation differences in each cancer. NGRs showed different methylation levels in each cancer, particularly COAD, PRAD, BRCA, KIRP, ESCA, PAAD, HNSC, UCEC, LUAD, BLCA, LIHC, LUSC, KIRC and THCA (Supplementary Figure 4A). Overall, the methylation level was found to be negatively associated with mRNA expression level in each cancer (Supplementary Figure 4B).

### Establishment of the NPS and its correlation with clinical outcomes

**Figure 3:**
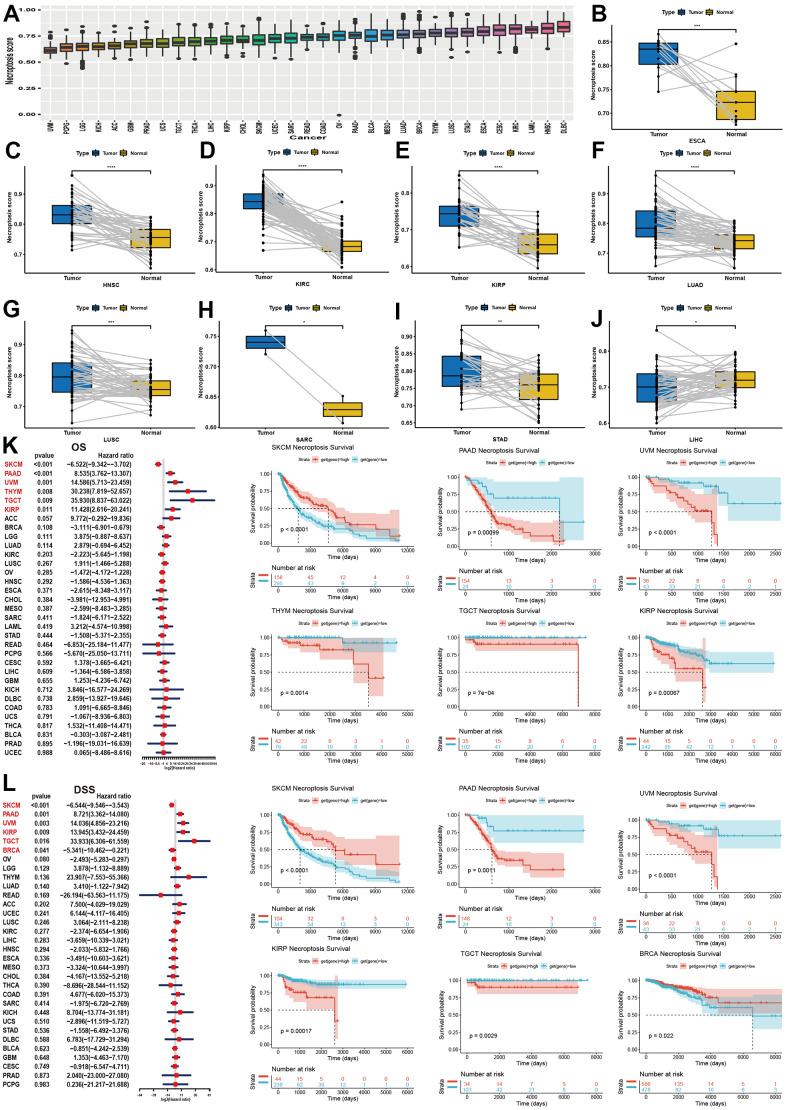
**Establishment of NPS and its clinical relevance in pan-cancer.** (**A**) Expression level of NPS in pan-cancer. (**B**–**J**) Significant expression levels of NPS between tumor and normal samples (HNSC, KIRC, KIRP, LUAD, LUSC, SARC, STAD, LIHC). (**K**) Association between NPS and overall survival in pan-caner using univariate cox regression (Left) and log-rank method (Right). (**L**) Association between NPS and disease-specific survival in pan-caner using univariate cox regression (Left) and log-rank method (Right).

We calculated the NPS of each sample in each cancer and found that DLBC exhibited the highest NPS and that the NPS of UVM was the lowest (Figure 3A). We then compared the NPSs in paired tumor and normal samples. The results showed that the NPS was significantly higher in the ESCA, HNSC, KIRC, KIRP, LUAD, LUSC, SARC, and STAD samples than the normal samples (Figure 3B–3I). However, the NPS was significantly lower in LIHC samples than in normal samples (Figure 3J).

Then, we investigated the correlation of NPS levels with four kinds of clinical outcomes.

For OS, the NPS was associated with a favorable prognosis in SKCM, while the NPS was found to be a risk factor for PAAD, UVM, THYM, TGCT and KIRP (Figure 3K). The Kaplan–Meier analysis indicated that a higher NPS indicated favorable OS in BRCA, DLBC, HNSC, KIRC, OV, READ, and STAD (Supplementary Figure 5). In contrast, the NPS was correlated with unfavorable survival in GBM, KICH, LGG, LUAD, and THYM (Supplementary Figure 5). For DSS (Figure 3L and Supplementary Figure 6), DFI (Supplementary Figures 7A, 8) and PFI (Supplementary Figures 7D–7L, 9), we found similar results. In summary, a high NPS was indicative of a good prognosis in SKCM, ESCA, OV, BRCA, and CHOL (Figure 3C), while a high NPS was an indicator of a poor prognosis in PAAD, KIRP, UVM, TGCT, BRCA and THYM.

### Correlations of necroptosis with signaling pathways and cell death in cancers

**Figure 4:**
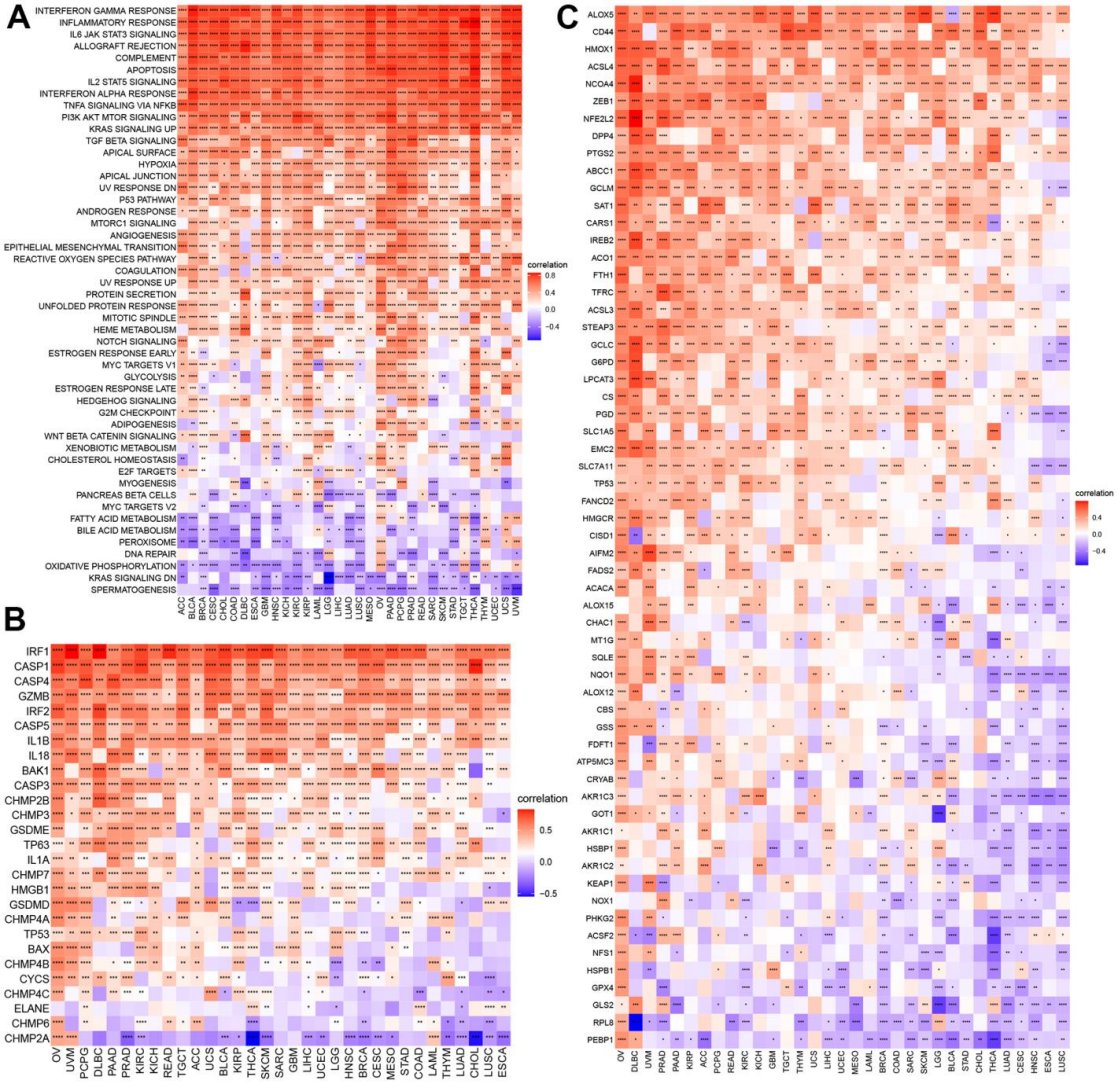
**GSVA of NPS and its correlations with cell death.** (**A**) GSVA of NPS. (**B**, **C**) Correlations of NPS with pyroptosis and ferroptosis regulators.

To explore the correlation of the NPS with signaling pathways, we performed a GSVA of necroptosis in cancers (Figure 4A). We found that the NPS was significantly and positively associated with the interferon gamma response, the inflammatory response, the IL6/JAK/STAT3 signaling pathway, allograft rejection, the complement system, the IL2/STAT5 signaling pathway, the interferon alpha response, TNF-alpha signaling via NFK-beta, and the TGF beta signaling pathway, which were mainly involved in immune response-related signaling. Some pathways were related to cell growth and apoptosis, including the p53 signaling pathway, and certain transduction pathways (PI3K/AKT/mTOR signaling pathway and KRAS signaling up) were positively correlated with the NPS. The NPS was significantly and negatively associated with MYC target V2 in 14 cancers, fatty acid metabolism and bile acid metabolism in 18 cancers, peroxisome in 14 cancers, DNA repair in 11 cancers, oxidative phosphorylation in 20 cancers, reduced KRAS signaling in 15 cancers, and spermatogenesis in 26 cancers. The MYC target V2 was related to tumorigenesis, and fatty and bile acid metabolism and oxidative phosphorylation, which were related to energy supply in tumors. KRAS signaling downregulation was part of a tumor inhibition pathway, and anticancer inhibitors targeting the KRAS signaling pathway have been developed [15]. DNA repair suppressed the antitumor effect of treatment. These results indicated that a high NPS indicates the inactivation of oncogenes, a reduction in energy supply, and the inhibition of tumor progression. However, the NPS showed positive associations with these signaling pathways in OV, TGCT, THYM, and UVM. These results indicated that NPS may exhibit different patterns in different cancers.

Necroptosis is a type of programmed cell death. We explored the correlations of NPS with pyroptosis, ferroptosis, and autophagy in each cancer. For pyroptosis, the NPS showed was significantly and positively associated with certain key molecules of pyroptosis, including CASP1, CAPS4, CASP3, IL18, IL1B, and GSDME [16], which are components of classical and nonclassical pyroptosis pathways (Figure 4B). Some molecules, such CHMP2A, showed negative associations with the NPS in cancers. ACLS4 is a key molecule that promotes ferroptosis [17], and the NPS was positively associated with ACLS4. Moreover, the NPS showed a negative association with GPX4 (an anti-ferroptosis-related molecule) in most cancers except PRAD, TGCT, and LAML (Figure 4C). In autophagy, LC3 is the key molecule [18]. We found that the NPS was negatively associated with LC3 (MAP1LC3A) expression in most cancers. However, a positive association was found with OV. Almost all autophagy genes were positively associated with the NPS in OV and DLBC (Supplementary Figure 10).

### Correlations of necroptosis with immune cell infiltration in cancers

**Figure 5:**
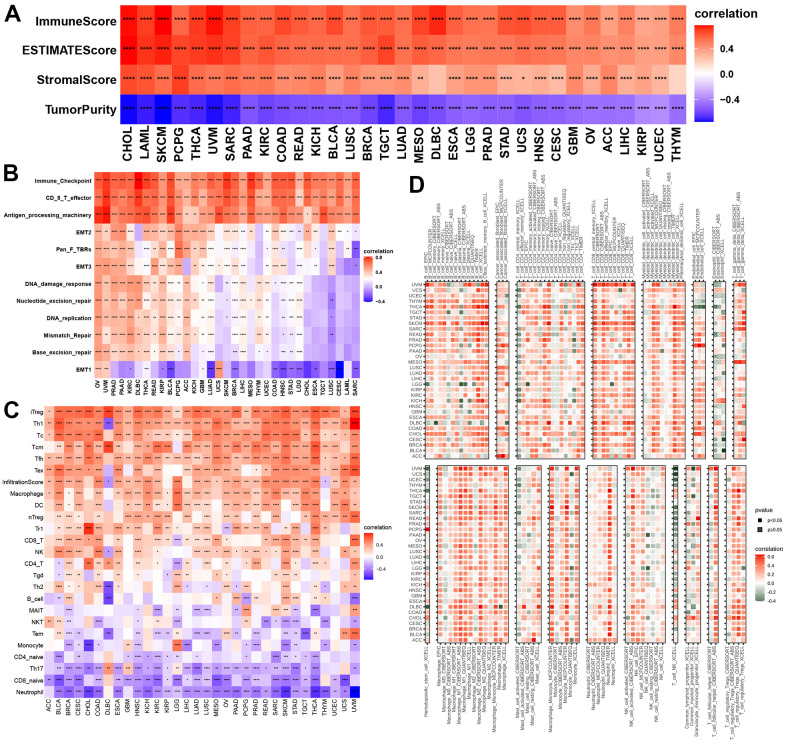
**Associations with NPS with immune status.** (**A**, **B**) Associations between NPS and tumor microenvironment in each cancer. (**C**, **D**) Associations between NPS and immune cell infiltrations in ImmuCell AI and TIMER2.

GSVA indicated that NPS was related to many immune-related signaling pathways. We further evaluated the correlations of NPS with immune status. Our results indicated that the NPS was positively associated with immune and ESTIMATE scores in all cancers and stromal scores in most cancers (Figure 5A). No significant correlations were found between the NPS and stromal score in DLBC and THYM. In contrast, the NPS was negatively associated with tumor purity in call cancers. Next, we assessed the correlations of the NPS with immune-related pathways, stromal/metastasis-related signaling pathways, and DNA repair-related pathways. These pathways were used to assess the tumor microenvironment [19]. We found that the NPS was positively associated with immune checkpoints, effector CD8 T cells, antigen-processing machinery, EMT2, pan-F-TBRs, and EMT3 in cancers (Figure 5B). These results indicated that the NPS potentially reflects the tumor microenvironment status.

Then, we evaluated the correlations of the NPS with 22 human immune cells in ImmuCellAI and TIMER2. Our results indicated that the NPS was positively associated with many immune cells including T cells (iTreg, Th1, Tc, Tfh, and Tex cells), cell infiltration score, macrophages, DCs, nTegs, Tr1 cells, and CD8 T cells, NK cells, and CD4 T cells in various cancers, while the NPS was negatively correlated to naïve CD 4 T cells, Th17 cells, CD8 naïve T cells, and neutrophils in all cancers (Figure 5C). Finally, we explored the correlations between the NPS and immune cells using the EPIC, CIBERSORT, ABS, QUANTISEQ, and XCELL methods. We found that the NPS was positively associated with M1 and M2 macrophages, mast cell resting, monocytes, neutrophils, activated NK-cells, naïve CD8+ and CD4+ T cells, memory B cells, and myeloid dendritic cells (Figure 5D). These results further proved that NPS shows promise as an index that can predict the immune status.

### Associations between necroptosis and molecular features and immune regulation

**Figure 6:**
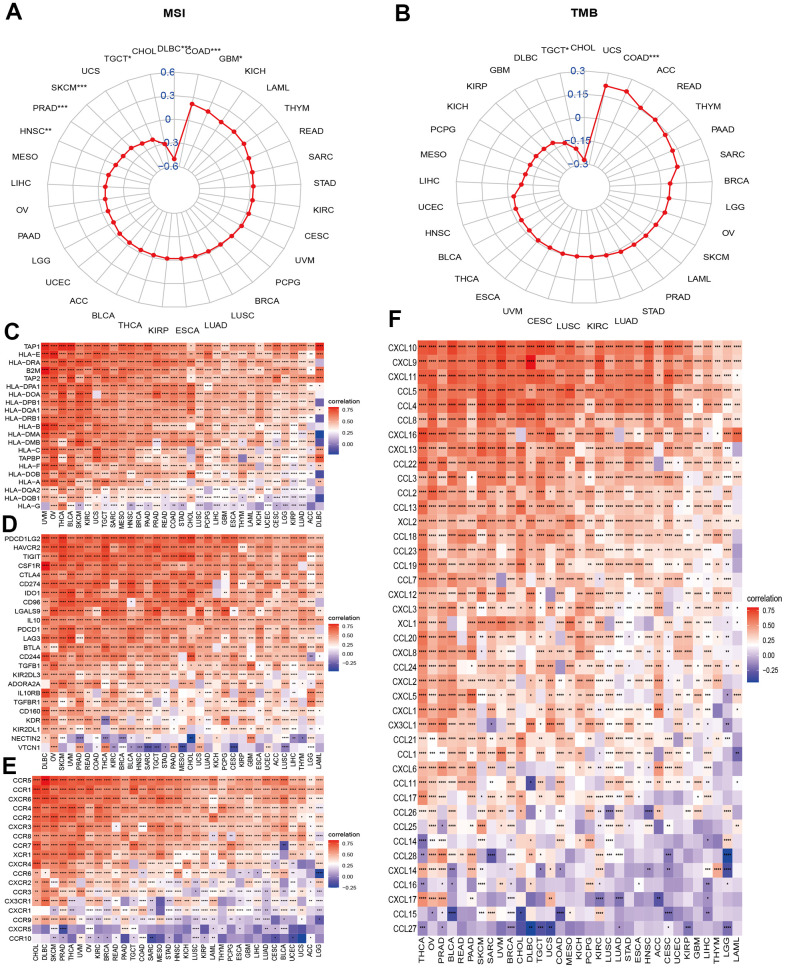
**Associations between NPS and molecular features and immune regulators.** (**A**, **B**) Associations between NPS with MSI and TMB in each cancer. (**C**–**F**) Associations between NPS and immune regulators: MHC genes, immune-regulated and checkpoint-related genes, chemokines, and chemokine receptors.

Microsatellite instability (MSI) and tumor mutation burden (TMB) are indicators of genome instability and mutation, respectively. We first evaluated the correlations of the NPS with MSI and TMB. The results indicated that the NPS was positively associated with MSI levels in COAD and GBM and was negatively associated with MSI levels in HNSC, PRAD, SKCM, TGCT, and DLBC (Figure 6A). NPS was positively associated with TBM in COAD but was negatively associated with TBM in TGCTs (Figure 6B).

Necroptosis is a process by which an organism attacks and kills the body’s cells by leveraging the immune system. We further explored the correlations of the NPS with certain important immune regulation gene sets. Major histocompatibility complex (MHC) molecules are involved in antigen recognition during immune responses. Human MHC is called human leukocyte antigen (HLA). The NPS was significantly and positively associated with HLA genes, including TAP1, HLA-E, HLA-DRA, B2M, TAP2, HLA-DPA1, and HLA-DOA in all cancers (Figure 6C). However, the NPS was negatively associated with HLA-G, an immune tolerance molecule that enables tumor cells to escape killing and lysis by NK cells and CTL cells, which is a previously unknown mechanism of tumor cell escape from immune surveillance. Next, we evaluated the correlations of the NPS with immune checkpoint and immune regulation genes in cancers. The results indicated that the NPS was highly correlated with immune checkpoint genes such as PD-L1 (CD274, Figure 6D), programmed cell death 1 ligand 2 (PDCD1LG2), tumor necrosis factor-related immune genes, and T lymphocyte-related immune genes (CD86, CD48, CD80, CD28, CD40, CD27, and CD70; Supplementary Figure 11). We also noticed that V-Set domain containing T-Cell activation inhibitor 1 (VTCN1) was negatively correlated with necroptosis levels in most cancers. These results suggested that the necroptosis level was closely related to most immune checkpoints. Chemokines and their receptors can induce the directional migration of immune cells, which is a necessary condition for the occurrence and completion of the immune response. We then evaluated the associations between necroptosis levels and chemokines and chemokine receptor genes in cancers (Figure 6E, 6F). Chemokines (CCR1, CCR2, CCR3, CCR4, CCR5, CCR6, CCR7, and CCR8) and receptor genes (CXCL1-17, CCL1-14, CCL16-26) were positively associated with necroptosis levels in various cancers (Figure 6). CCL27 and CCL15 showed negative correlations with necroptosis levels in certain cancers. These associations further indicated that the necroptosis level was highly correlated with immune regulation.

### Necroptosis and immunotherapy

**Figure 7:**
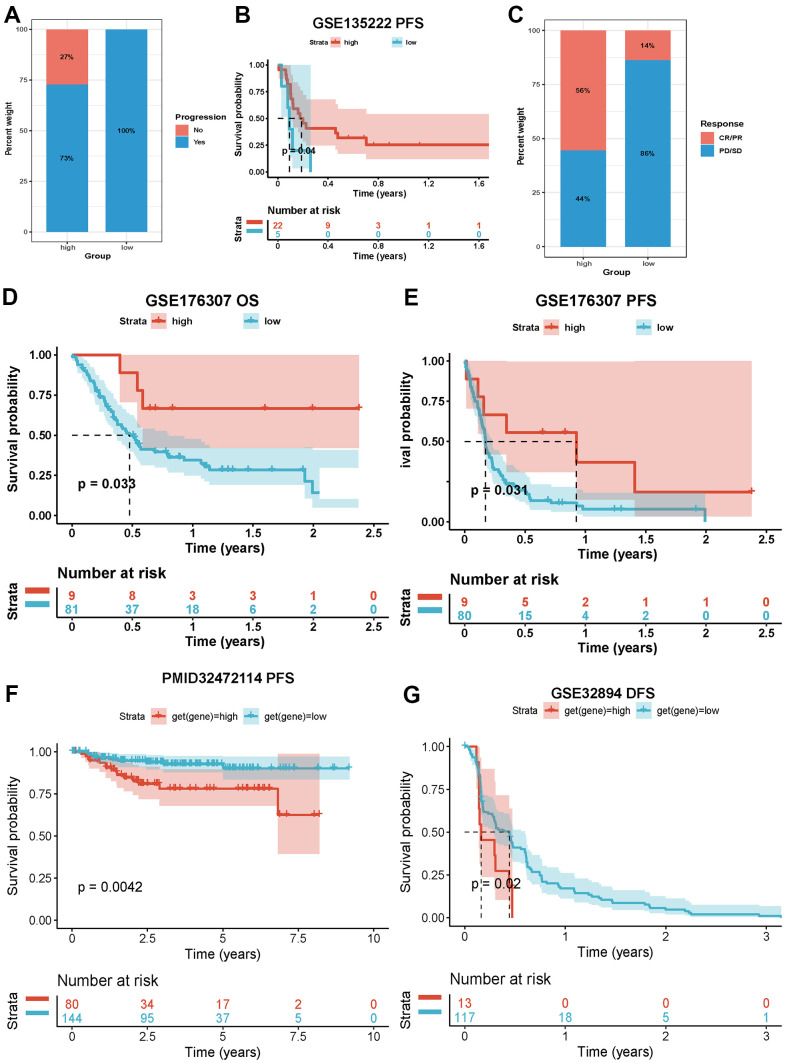
**Impacts of NPS on immunotherapy.** (**A**, **B**) Associations between NPS and immune response and PFS in advanced non-small cell lung carcinoma patients who receiving immunotherapy. (**C**–**E**) Associations between NPS and immune response, OS and PFS in metastatic urothelial cancer patients who receiving immunotherapy. (**F**) Associations between NPS and PFS in KIRC patients who receiving immunotherapy. (**G**) Associations between NPS and PFS in urothelium carcinoma patients who receiving immunotherapy.

Our comprehensive analysis revealed that necroptosis was involved in immune infiltration and immune regulation in cancers. We then evaluated the effect of necroptosis on immunotherapy. In bladder cancer (GSE13507), we found that patients with a high NPS exhibited a lower progression ratio than those with a low NPS (Figure 7A), and the Kaplan–Meier curves indicated that patients with a high NPS showed favorable progression-free survival (P<0.04, Figure 7B). In metastatic urothelial cancer (GSE176307), the CR/PR response rate of immunotherapy was higher in the high-NPS group than in the low-NPS group (56% vs. 14%, P<0.001, Figure 7C). The Kaplan–Meier analysis indicated that the high-NPS group presented with a higher OS (*P*=0.033, Figure 7D) and PFS (P=0.031, Figure 7E). However, in KIRC (PMID: 32472114) and urothelium carcinoma (GSE32894), the high-NPS group showed a worsened PFS (KIRC: P=0.0042, Figure 7F) and disease-free survival (urothelium carcinoma: P=0.02, Figure 7G). These results indicated that NPS can potentially be a marker for predicting the prognosis of patients who receive immunotherapy.

### Necroptosis and chemotherapy sensitivity

**Figure 8:**
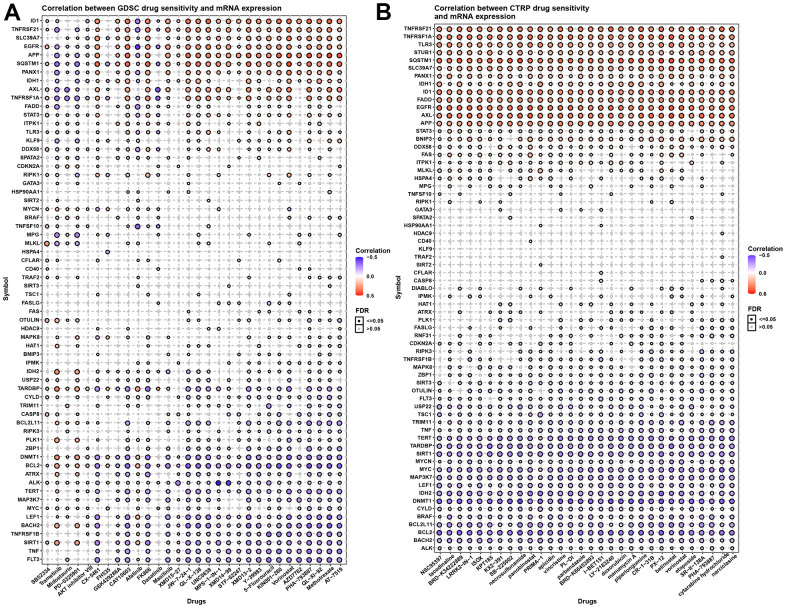
**Necroptosis and chemotherapy sensitivity.** (**A**) Necroptosis regulators and GDSC drug sensitivity. (**B**) Necroptosis regulators and CTRP drug sensitivity.

A previous study has reported that chemotherapy can exert a treatment effect by inducing necroptosis. We therefore evaluated the correlation between necroptosis regulators and certain small-molecule compounds (30 kinds of drugs) in cancers using the GDSC and CTRP databases (Figure 8A, 8B). We observed that IDH1, TNFRSF21, SLC39A7, EGFR, APP, SQSTM1, PANX1, IDH1, AXL, TNFRSF1A, STAT3, STUB1, TLR3, and FADDBNIP3 induced chemotherapy resistance to common drugs. In contrast, ALK, BACH2, BCL2L11, BRAF, CYLT, DNMT1, IDH2, LEF1, MAP3K7, MYC, MTCN, SIRT1, TARDBP, TERT, TSC1, USP22, and FLT3 enhance the sensitivity of these drugs in cancers. More details can be found in Supplementary Tables 3, 4. These results provide potential therapeutic targets for chemotherapy in cancers.

## DISCUSSION

The occurrence of cancer is closely related to cell death [20, 21]. In addition to apoptosis, necroptosis has also been related to the development of cancer [22]. However, studies have also demonstrated that necroptosis plays a dual role in cancer progression and regression [23, 24]. Among these roles, targeting necroptosis-related proteins exerts a dual impact on tumor initiation and progression. It is generally believed that dysfunctional necroptosis is related to the occurrence and development of tumors. For example, the expression of RIP3 is significantly downregulated in patients with acute myeloid leukemia (AML). A reduction in RIP3 expression reduces hematopoietic cell death, which is associated with the development of AML [25]. Another study has reported that genetic defects in RIP3 converted FLT3-ITD- and RUNXETO-driven myeloproliferative AML in mice by increasing the accumulation of leukemia-initiating cells [26]. Furthermore, low expression of MLKL was associated with reduced overall survival in colon cancer patients after surgery [27]. MLKL is also downregulated in pancreatic and cervical squamous cell carcinomas, where low levels of MLKL in plasma predict poor prognosis in pancreatic and ovarian cancers [28, 29]. Previous study also explored the role of necroptosis in single cancer types. Zhao developed and validated a necroptosis-related lncRNA model used for predicting prognosis in gastric cancer [30]. Similarly, Liu also explored the role of necroptosis-related lncRNAs and found necroptosis-related lncRNAs had an important function, and the prognosis of patients with colon cancer can be predicted by six necroptosis-related lncRNAs [31]. Using 10 necroptosis genes, Wu developed a novel necroptosis-related gene signature for predicting the prognosis of pancreatic adenocarcinoma [32]. The other study evaluated the expression of key molecules in necroptosis and their association with clinical features and prognosis in NSCLC [33]. Different from previous studies, in our study, we found that the established necroptosis score (NPS) was associated with a favorable prognosis in SKCM, while the NPS was a risk factor for PAAD, UVM, THYM, TGCT and KIRP. The Kaplan–Meier analysis indicated that a higher NPS indicated favorable OS in BRCA, DLBC, HNSC, KIRC, OV, READ, and STAD (Supplementary Figure 5). In contrast, the NPS was correlated with unfavorable survival in GBM, KICH, LGG, LUAD, and THYM. Altogether, this information provides research directions for studying necrotic proteins in tumor development, recent studies have found that cell resistance to necroptosis is often mediated by oncogenes, suggesting that escape from necroptosis, similar to escape from apoptosis, may be a potential hallmark of tumors [34].

We found that NPS was positively associated with immune-related and inflammation-associated signaling pathways. This result suggested that the function of necroptosis may be associated with immunity and inflammation in cancers. Necroptosis is a type of programmed cell death. We explored the correlations of the NPS with pyroptosis, ferroptosis, and autophagy in each cancer. We found that necroptosis was closely associated with certain key molecules involved in pyroptosis, ferroptosis, and autophagy. Our findings indicated a complex and close relationship among necroptosis, pyroptosis, ferroptosis and autophagy. Previous studies have found that caspase-8 can act as a bridge between different types of cell death and play key functions in the apoptosis and necroptosis pathways [35]. In addition, researchers have recently discovered another link between pyroptosis and apoptosis. In macrophages, activation of caspase-1 redirected cell fate toward caspase-3-, caspase-9- and BID-dependent apoptosis in the absence of GSDMD [36]. RNA viruses induced NLRP3 activation and subsequent IL-1β release in a RIPK1/RIPK3-dependent and MLKL-independent manner, which may be another example of an immune response linking different cell death pathways [37]. TAK1 was found to form a complex with RIPK1, FADD, and caspase-8 downstream of TNFR or TLR and has recently emerged as another survival-promoting regulator of cell death, further blurring the lines between cell death pathways [38]. In addition, all types of cell death are accompanied by inflammation, which is a link among these types of cell death [39–42]. Overall, the current study highlights the tight cross-regulation between cell death and shows that when one pathway is compromised, bridges between pathways coordinate cell death.

We then explored the correlation of necroptosis levels with the tumor microenvironment and immune cell infiltration. We found that necroptosis was markedly and positively associated with immune, ESTIMATE, and stromal scores in cancers but negatively associated with tumor purity. Furthermore, we found that necroptosis was positively associated with macrophages, neutrophils, B cells, and CD8 T cells in cancers. It has been reported that antitumor immunity can be obtained by activation of CD8+ T cells through antigen cross-linking [43]. This may be caused molecules released from dying cells passing through antigen-presenting cells, such as dendritic cells. Activation of tumor cell necroptosis enhances antitumor immunity [44, 45]. Therefore, targeting necrosis to induce antitumor immunity is a viable strategy, especially when apoptosis is blocked. To explore whether necroptosis levels and guide immunotherapy, we evaluated the correlations of necroptosis levels with immune checkpoint gene expression and found that necroptosis levels were highly correlated with immune checkpoint genes such as PD-L1, programmed cell death 1 ligand 2, tumor necrosis factor-related immune genes, and T lymphocyte-related immune genes. Next, we evaluated the effect of necroptosis levels on immunotherapy. In bladder cancer and metastatic urothelial cancer, we found that patients with high necroptosis levels had a better prognosis than those with low necroptosis levels. However, in KIRC and urothelium carcinoma, the high necroptosis level group presented with worsened PFS and DFS. We also evaluated the correlations of necroptosis regulators and certain small-molecule compounds in cancers and identified chemotherapy sensitivity and resistance-relevant small-molecule compounds. The differences in prognosis in different cancers further indicated the dual role played by necroptosis in cancers. The primary limitation of this study is lack of experiments *in vivo* and *vitro*. The present findings need to be validated by molecular biology experiments. Our study found that necroptosis may involve in immune regulation and drug sensitivity. The future should focus on the role of necroptosis in immunity treatment and chemotherapy in various cancers.

Our results indicated that the necroptosis score can be considered a prognostic factor in multiple cancer types. Further analysis suggested that necroptosis can affect the tumor microenvironment and immunity regulation, and the effect of necroptosis on immunity differs by tumor type. The process of activating necroptosis involves multiple signaling pathways, and crosstalk is involved between necroptosis and other cell death types. Furthermore, the necroptosis level may be a biomarker for immunotherapy in multiple cancers. To the best of our knowledge, this is the first study to systematically explore necroptosis across cancers, and our results not only highlight the effect of necroptosis on immune regulation and immunotherapy but also shows important significance for the identification of tumor therapeutic targets, development of new target drugs and study of tumor drug resistance mechanisms.

## Supplementary Material

Supplementary Figures

Supplementary Tables 1 and 2

Supplementary Table 3

Supplementary Table 4

## References

[1] Carson DA, Ribeiro JM. Apoptosis and disease. Lancet. 1993; 341:1251–4. 10.1016/0140-6736(93)91154-e 8098400

[2] Majno G, Joris I. Apoptosis, oncosis, and necrosis. An overview of cell death. Am J Pathol. 1995; 146:3–15. 7856735 PMC1870771

[3] Degterev A, Huang Z, Boyce M, Li Y, Jagtap P, Mizushima N, Cuny GD, Mitchison TJ, Moskowitz MA, Yuan J. Chemical inhibitor of nonapoptotic cell death with therapeutic potential for ischemic brain injury. Nat Chem Biol. 2005; 1:112–9. 10.1038/nchembio711 16408008

[4] Weinlich R, Oberst A, Beere HM, Green DR. Necroptosis in development, inflammation and disease. Nat Rev Mol Cell Biol. 2017; 18:127–36. 10.1038/nrm.2016.149 27999438

[5] Pasparakis M, Vandenabeele P. Necroptosis and its role in inflammation. Nature. 2015; 517:311–20. 10.1038/nature14191 25592536

[6] Yan J, Wan P, Choksi S, Liu ZG. Necroptosis and tumor progression. Trends Cancer. 2022; 8:21–7. 10.1016/j.trecan.2021.09.003 34627742 PMC8702466

[7] Özdemir BH. Tumor Microenvironment: Necroptosis Switches the Subtype of Liver Cancer While Necrosis Promotes Tumor Recurrence and Progression. Exp Clin Transplant. 2022. [Epub ahead of print]. 10.6002/ect.2021.0457 35297332

[8] Niu X, Chen L, Li Y, Hu Z, He F. Ferroptosis, necroptosis, and pyroptosis in the tumor microenvironment: Perspectives for immunotherapy of SCLC. Semin Cancer Biol. 2022; S1044-579X:00065–7. 10.1016/j.semcancer.2022.03.009 35288298

[9] Snyder AG, Hubbard NW, Messmer MN, Kofman SB, Hagan CE, Orozco SL, Chiang K, Daniels BP, Baker D, Oberst A. Intratumoral activation of the necroptotic pathway components RIPK1 and RIPK3 potentiates antitumor immunity. Sci Immunol. 2019; 4:eaaw2004. 10.1126/sciimmunol.aaw2004 31227597 PMC6831211

[10] Scarpitta A, Hacker UT, Büning H, Boyer O, Adriouch S. Pyroptotic and Necroptotic Cell Death in the Tumor Microenvironment and Their Potential to Stimulate Anti-Tumor Immune Responses. Front Oncol. 2021; 11:731598. 10.3389/fonc.2021.731598 34490126 PMC8417056

[11] Liu Y, Chen Q, Zhu Y, Wang T, Ye L, Han L, Yao Z, Yang Z. Non-coding RNAs in necroptosis, pyroptosis and ferroptosis in cancer metastasis. Cell Death Discov. 2021; 7:210. 10.1038/s41420-021-00596-9 34381023 PMC8358062

[12] Barbie DA, Tamayo P, Boehm JS, Kim SY, Moody SE, Dunn IF, Schinzel AC, Sandy P, Meylan E, Scholl C, Fröhling S, Chan EM, Sos ML, et al. Systematic RNA interference reveals that oncogenic KRAS-driven cancers require TBK1. Nature. 2009; 462:108–12. 10.1038/nature08460 19847166 PMC2783335

[13] Zeng D, Li M, Zhou R, Zhang J, Sun H, Shi M, Bin J, Liao Y, Rao J, Liao W. Tumor Microenvironment Characterization in Gastric Cancer Identifies Prognostic and Immunotherapeutically Relevant Gene Signatures. Cancer Immunol Res. 2019; 7:737–50. 10.1158/2326-6066.CIR-18-0436 30842092

[14] Braun DA, Hou Y, Bakouny Z, Ficial M, Sant’ Angelo M, Forman J, Ross-Macdonald P, Berger AC, Jegede OA, Elagina L, Steinharter J, Sun M, Wind-Rotolo M, et al. Interplay of somatic alterations and immune infiltration modulates response to PD-1 blockade in advanced clear cell renal cell carcinoma. Nat Med. 2020; 26:909–18. 10.1038/s41591-020-0839-y 32472114 PMC7499153

[15] Kim HJ, Lee HN, Jeong MS, Jang SB. Oncogenic KRAS: Signaling and Drug Resistance. Cancers (Basel). 2021; 13:5599. 10.3390/cancers13225599 34830757 PMC8616169

[16] Fang Y, Tian S, Pan Y, Li W, Wang Q, Tang Y, Yu T, Wu X, Shi Y, Ma P, Shu Y. Pyroptosis: A new frontier in cancer. Biomed Pharmacother. 2020; 121:109595. 10.1016/j.biopha.2019.109595 31710896

[17] Mou Y, Wang J, Wu J, He D, Zhang C, Duan C, Li B. Ferroptosis, a new form of cell death: opportunities and challenges in cancer. J Hematol Oncol. 2019; 12:34. 10.1186/s13045-019-0720-y 30925886 PMC6441206

[18] Levy JMM, Towers CG, Thorburn A. Targeting autophagy in cancer. Nat Rev Cancer. 2017; 17:528–42. 10.1038/nrc.2017.53 28751651 PMC5975367

[19] Arneth B. Tumor Microenvironment. Medicina (Kaunas). 2019; 56:15. 10.3390/medicina56010015 31906017 PMC7023392

[20] Qiu Y, Li P, Ji C. Cell Death Conversion under Hypoxic Condition in Tumor Development and Therapy. Int J Mol Sci. 2015; 16:25536–51. 10.3390/ijms161025536 26512660 PMC4632814

[21] Martinez-Lostao L, de Miguel D, Al-Wasaby S, Gallego-Lleyda A, Anel A. Death ligands and granulysin: mechanisms of tumor cell death induction and therapeutic opportunities. Immunotherapy. 2015; 7:883–2. 10.2217/imt.15.56 26314314

[22] Lee SY, Ju MK, Jeon HM, Jeong EK, Lee YJ, Kim CH, Park HG, Han SI, Kang HS. Regulation of Tumor Progression by Programmed Necrosis. Oxid Med Cell Longev. 2018; 2018:3537471. 10.1155/2018/3537471 29636841 PMC5831895

[23] Liu ZG, Jiao D. Necroptosis, tumor necrosis and tumorigenesis. Cell Stress. 2019; 4:1–8. 10.15698/cst2020.01.208 31922095 PMC6946014

[24] Sakanashi F, Shintani M, Tsuneyoshi M, Ohsaki H, Kamoshida S. Apoptosis, necroptosis and autophagy in colorectal cancer: Associations with tumor aggressiveness and p53 status. Pathol Res Pract. 2019; 215:152425. 10.1016/j.prp.2019.04.017 31097354

[25] Nugues AL, El Bouazzati H, Hétuin D, Berthon C, Loyens A, Bertrand E, Jouy N, Idziorek T, Quesnel B. RIP3 is downregulated in human myeloid leukemia cells and modulates apoptosis and caspase-mediated p65/RelA cleavage. Cell Death Dis. 2014; 5:e1384. 10.1038/cddis.2014.347 25144719 PMC4454320

[26] Höckendorf U, Yabal M, Herold T, Munkhbaatar E, Rott S, Jilg S, Kauschinger J, Magnani G, Reisinger F, Heuser M, Kreipe H, Sotlar K, Engleitner T, et al. RIPK3 Restricts Myeloid Leukemogenesis by Promoting Cell Death and Differentiation of Leukemia Initiating Cells. Cancer Cell. 2016; 30:75–91. 10.1016/j.ccell.2016.06.002 27411587

[27] Li X, Guo J, Ding AP, Qi WW, Zhang PH, Lv J, Qiu WS, Sun ZQ. Association of Mixed Lineage Kinase Domain-Like Protein Expression With Prognosis in Patients With Colon Cancer. Technol Cancer Res Treat. 2017; 16:428–34. 10.1177/1533034616655909 27432118 PMC5616063

[28] Seldon CS, Colbert LE, Hall WA, Fisher SB, Yu DS, Landry JC. Chromodomain-helicase-DNA binding protein 5, 7 and pronecrotic mixed lineage kinase domain-like protein serve as potential prognostic biomarkers in patients with resected pancreatic adenocarcinomas. World J Gastrointest Oncol. 2016; 8:358–65. 10.4251/wjgo.v8.i4.358 27096031 PMC4824714

[29] Ruan J, Mei L, Zhu Q, Shi G, Wang H. Mixed lineage kinase domain-like protein is a prognostic biomarker for cervical squamous cell cancer. Int J Clin Exp Pathol. 2015; 8:15035–8. 26823841 PMC4713627

[30] Zhao Z, Liu H, Zhou X, Fang D, Ou X, Ye J, Peng J, Xu J. Necroptosis-Related lncRNAs: Predicting Prognosis and the Distinction between the Cold and Hot Tumors in Gastric Cancer. J Oncol. 2021; 2021:6718443. 10.1155/2021/6718443 34790235 PMC8592775

[31] Liu L, Huang L, Chen W, Zhang G, Li Y, Wu Y, Xiong J, Jie Z. Comprehensive Analysis of Necroptosis-Related Long Noncoding RNA Immune Infiltration and Prediction of Prognosis in Patients With Colon Cancer. Front Mol Biosci. 2022; 9:811269. 10.3389/fmolb.2022.811269 35237659 PMC8883231

[32] Wu Z, Huang X, Cai M, Huang P, Guan Z. Novel necroptosis-related gene signature for predicting the prognosis of pancreatic adenocarcinoma. Aging (Albany NY). 2022; 14:869–91. 10.18632/aging.203846 35077391 PMC8833111

[33] Park JE, Lee JH, Lee SY, Hong MJ, Choi JE, Park S, Jeong JY, Lee EB, Choi SH, Lee YH, Seo HW, Yoo SS, Lee J, et al. Expression of key regulatory genes in necroptosis and its effect on the prognosis in non-small cell lung cancer. J Cancer. 2020; 11:5503–10. 10.7150/jca.46172 32742497 PMC7391199

[34] Najafov A, Zervantonakis IK, Mookhtiar AK, Greninger P, March RJ, Egan RK, Luu HS, Stover DG, Matulonis UA, Benes CH, Yuan J. BRAF and AXL oncogenes drive RIPK3 expression loss in cancer. PLoS Biol. 2018; 16:e2005756. 10.1371/journal.pbio.2005756 30157175 PMC6114281

[35] Newton K, Wickliffe KE, Maltzman A, Dugger DL, Reja R, Zhang Y, Roose-Girma M, Modrusan Z, Sagolla MS, Webster JD, Dixit VM. Activity of caspase-8 determines plasticity between cell death pathways. Nature. 2019; 575:679–82. 10.1038/s41586-019-1752-8 31723262

[36] Tsuchiya K, Nakajima S, Hosojima S, Thi Nguyen D, Hattori T, Manh Le T, Hori O, Mahib MR, Yamaguchi Y, Miura M, Kinoshita T, Kushiyama H, Sakurai M, et al. Caspase-1 initiates apoptosis in the absence of gasdermin D. Nat Commun. 2019; 10:2091. 10.1038/s41467-019-09753-2 31064994 PMC6505044

[37] Wang X, Jiang W, Yan Y, Gong T, Han J, Tian Z, Zhou R. RNA viruses promote activation of the NLRP3 inflammasome through a RIP1-RIP3-DRP1 signaling pathway. Nat Immunol. 2014; 15:1126–33. 10.1038/ni.3015 25326752

[38] Zheng Z, Deng W, Bai Y, Miao R, Mei S, Zhang Z, Pan Y, Wang Y, Min R, Deng F, Wu Z, Li W, Chen P, et al. The Lysosomal Rag-Ragulator Complex Licenses RIPK1 and Caspase-8-mediated Pyroptosis by *Yersinia*. Science. 2021; 372:eabg0269. 10.1126/science.abg0269 35058659 PMC8769499

[39] Peltzer N, Walczak H. Cell Death and Inflammation - A Vital but Dangerous Liaison. Trends Immunol. 2019; 40:387–402. 10.1016/j.it.2019.03.006 31003931

[40] Grivennikov SI, Greten FR, Karin M. Immunity, inflammation, and cancer. Cell. 2010; 140:883–99. 10.1016/j.cell.2010.01.025 20303878 PMC2866629

[41] Singh R, Mishra MK, Aggarwal H. Inflammation, Immunity, and Cancer. Mediators Inflamm. 2017; 2017:6027305. 10.1155/2017/6027305 29234189 PMC5695028

[42] Newton K, Dixit VM. Signaling in innate immunity and inflammation. Cold Spring Harb Perspect Biol. 2012; 4:a006049. 10.1101/cshperspect.a006049 22296764 PMC3282411

[43] St Paul M, Saibil SD, Han S, Israni-Winger K, Lien SC, Laister RC, Sayad A, Penny S, Amaria RN, Haydu LE, Garcia-Batres CR, Kates M, Mulder DT, et al. Coenzyme A fuels T cell anti-tumor immunity. Cell Metab. 2021; 33:2415–27.e6. 10.1016/j.cmet.2021.11.010 34879240

[44] Shibata K, Omahdi Z, Yamasaki S. Necroptosis DAMPens anti-tumor immunity. Cell Death Discov. 2016; 2:16033. 10.1038/cddiscovery.2016.33 27551523 PMC4979415

[45] Meng MB, Wang HH, Cui YL, Wu ZQ, Shi YY, Zaorsky NG, Deng L, Yuan ZY, Lu Y, Wang P. Necroptosis in tumorigenesis, activation of anti-tumor immunity, and cancer therapy. Oncotarget. 2016; 7:57391–413. 10.18632/oncotarget.10548 27429198 PMC5302997

