# Supplementary material for: Pan-cancer analysis of a novel indicator of necroptosis with its application in human cancer
Source: Aging (Albany NY). 2022 Sep 26;14(18):7587–616. doi: 10.18632/aging.204307 (PMC9550240; doi:10.18632/aging.204307)
Supplement: Supplementary Figures [file aging-14-204307-s001.pdf]

SUPPLEMENTARY FIGURES

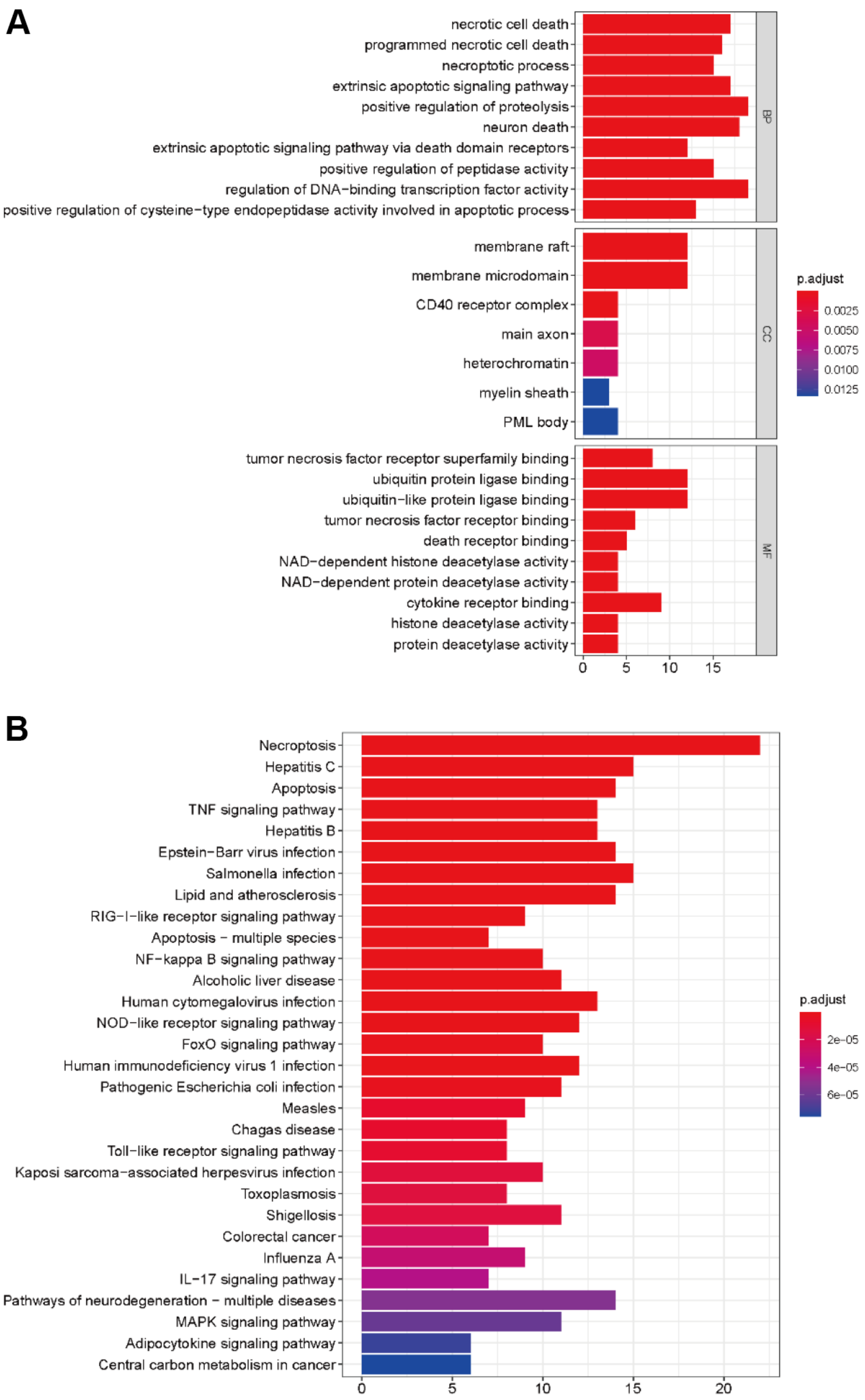

Supplementary Figure 1. Functional and pathways enrichment of necroptosis regulators. (**A**) GO enrichment. (**B**) KEGG pathways enrichment.

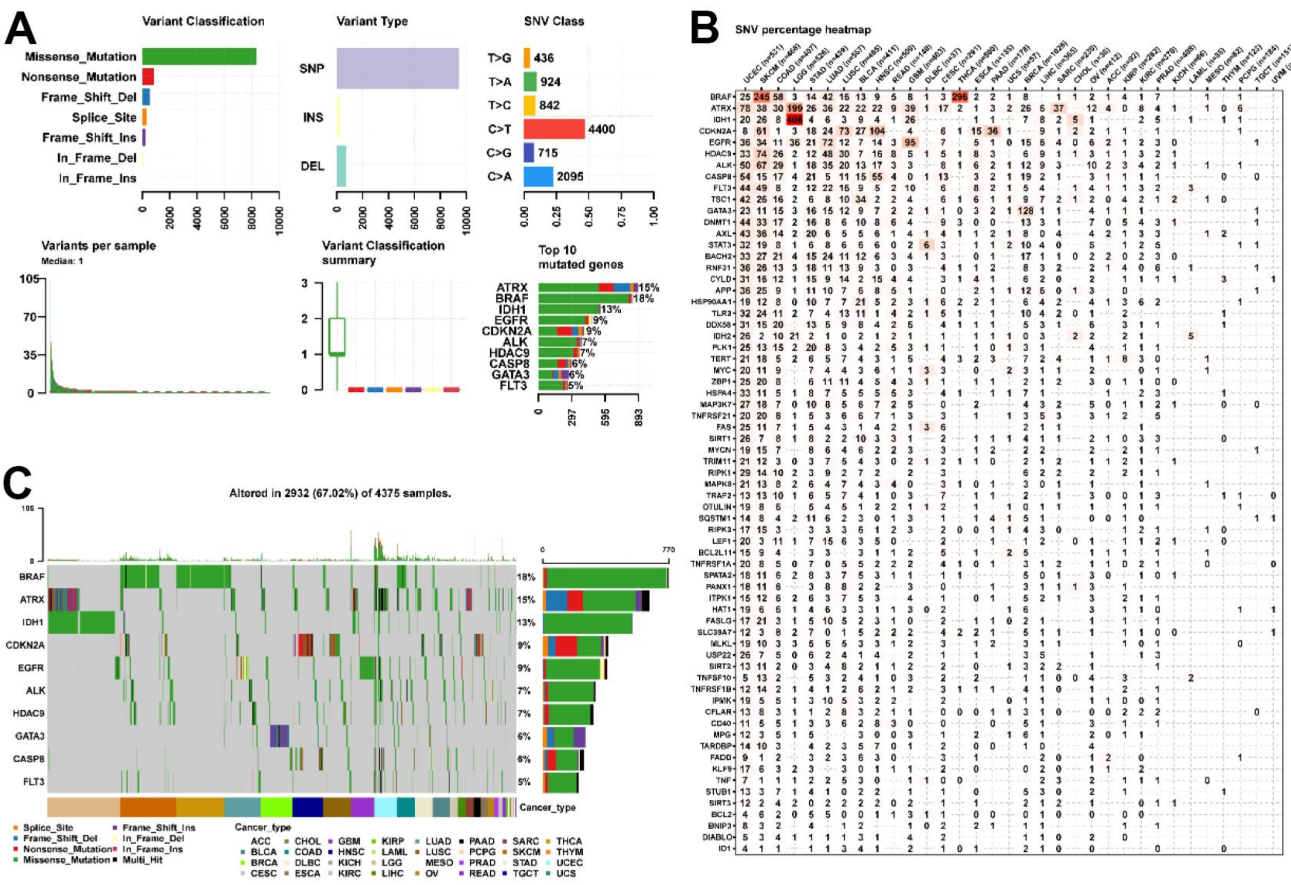

Supplementary Figure 2. Gene alterations of necroptosis regulators. (**A**) Variant classification and types of necroptosis in pan-cancer. (**B**) single nucleotide variations frequencies of necroptosis in pan-cancer. (**C**) Top gene of necroptosis alterations in pan-cancer.

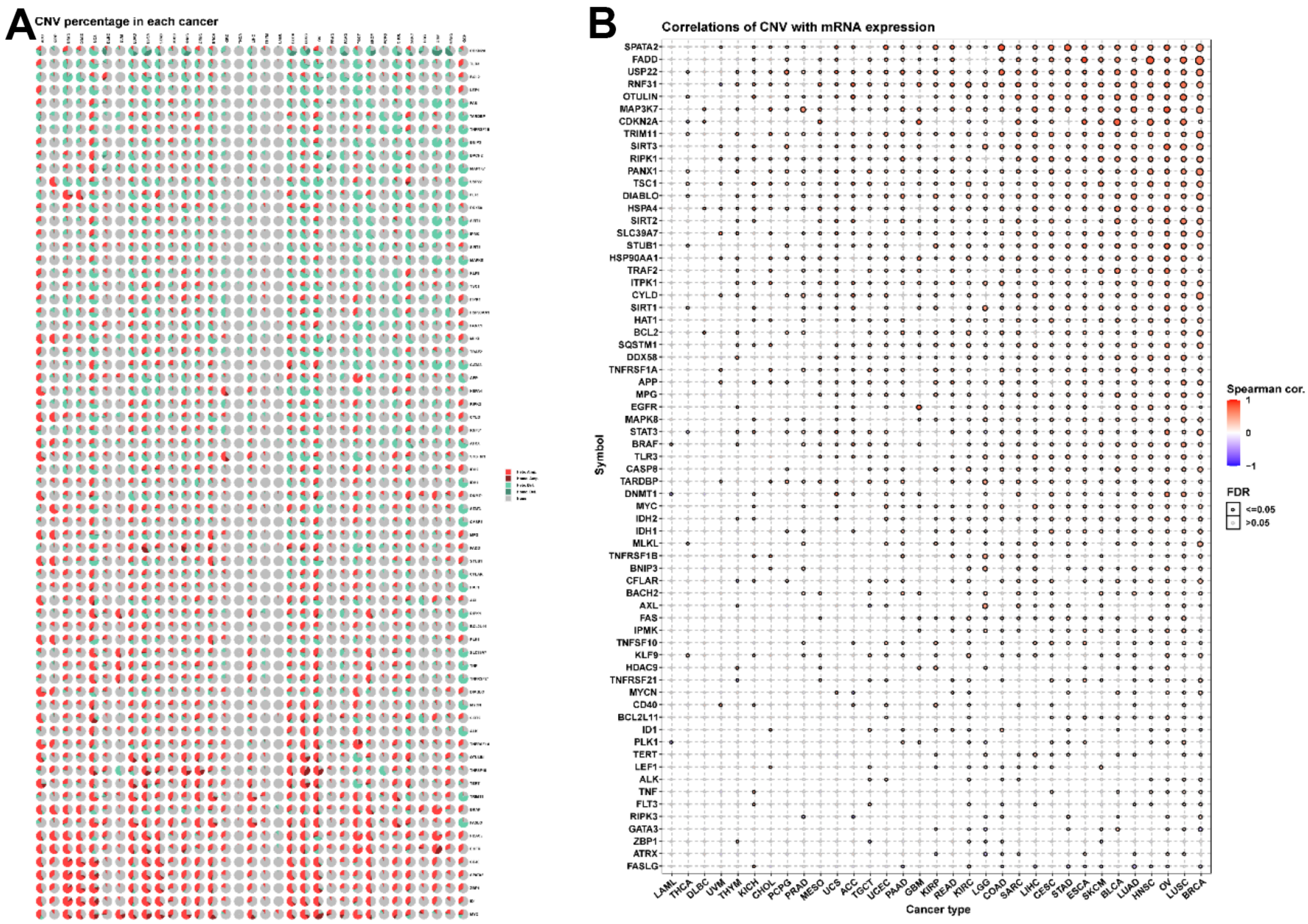

Supplementary Figure 3. Copy number variations of necroptosis in pan-cancer. (**A**) copy number variation percentage of necroptosis in each cancer. (**B**) Correlations of copy number with mRNA expression.

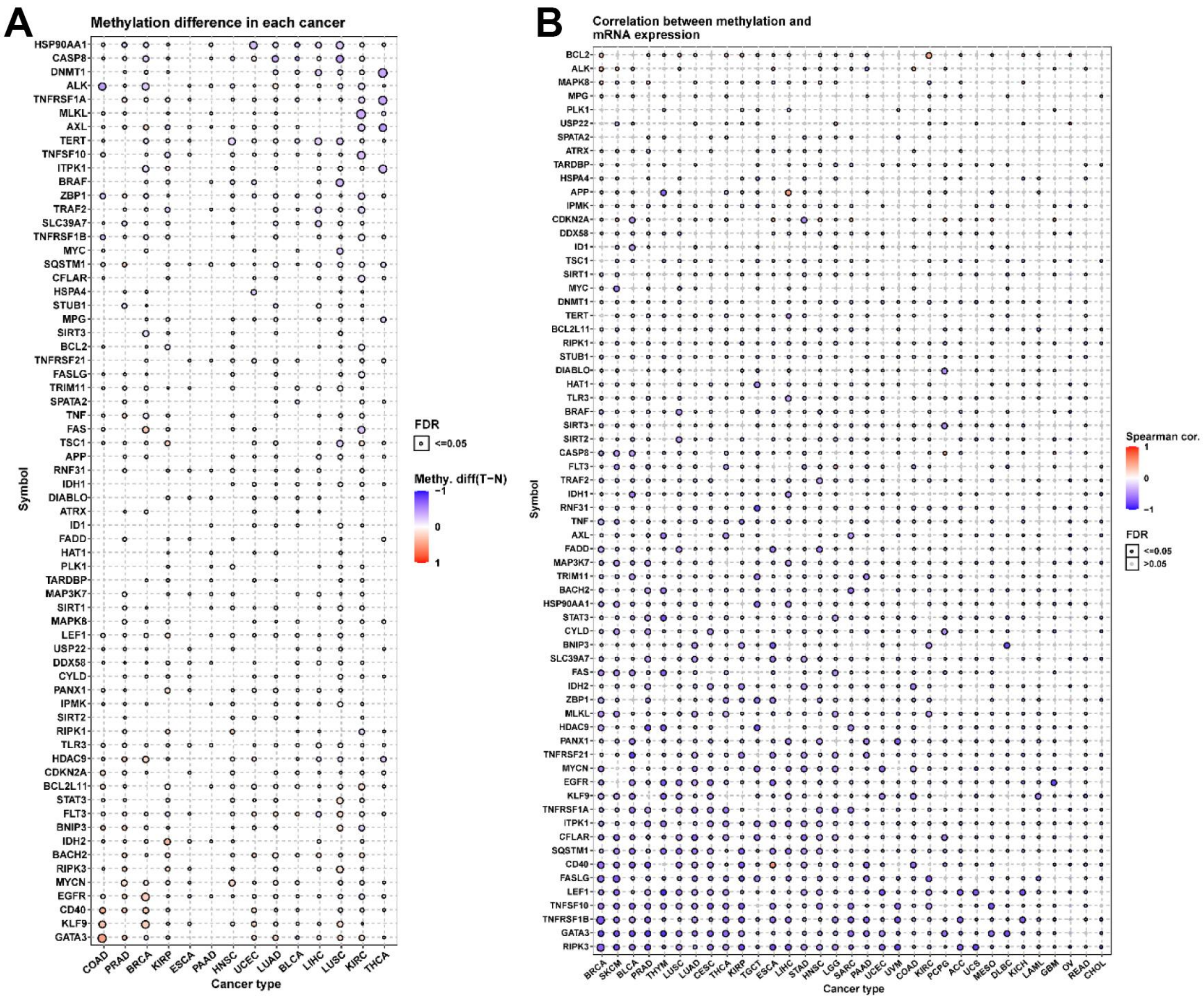

Supplementary Figure 4. Methylation profiling of necroptosis regulators in cancers. (**A**) Methylations difference in each cancer. (**B**) Correlations between methylation and mRNA expression. Pan-caner using univariate cox regression (left) and log-rank method (Right).

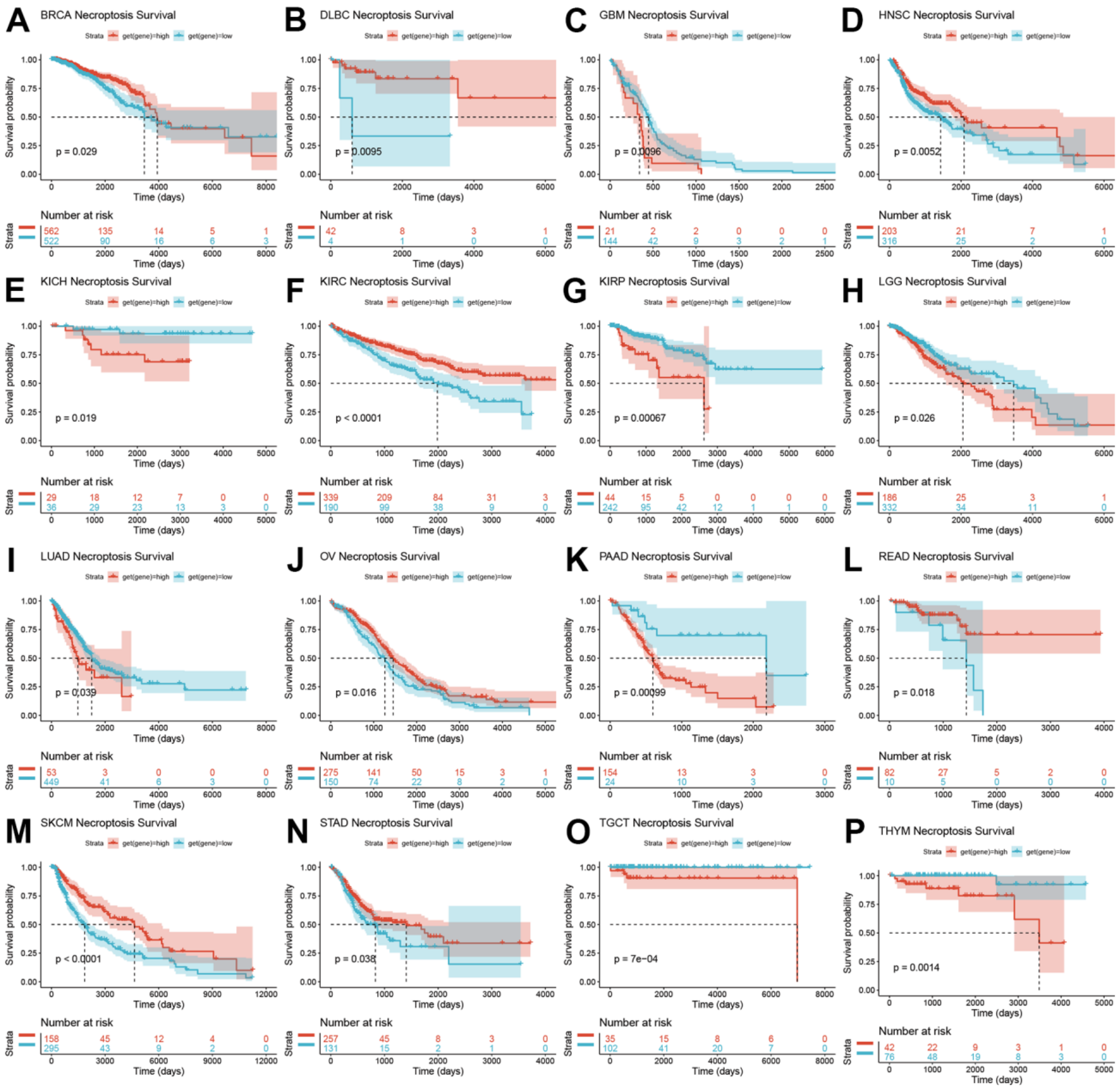

Supplementary Figure 5. Associations between NPS and disease-specific survival in pan-cancer using log-rank method. (**A**) BRCA. (**B**) GBM. (**C**) HNSC. (**D**) KICH. (**E**) KIRC. (**F**) KIRP. (**G**) LGG. (**H**) LUSC. (**I**) MESO. (**J**) OV. (**K**) PAAD. (**L**) SKCM. (**M**) STAD. (**N**) TGCT. (**O**) THYM (**P**) UCEC.

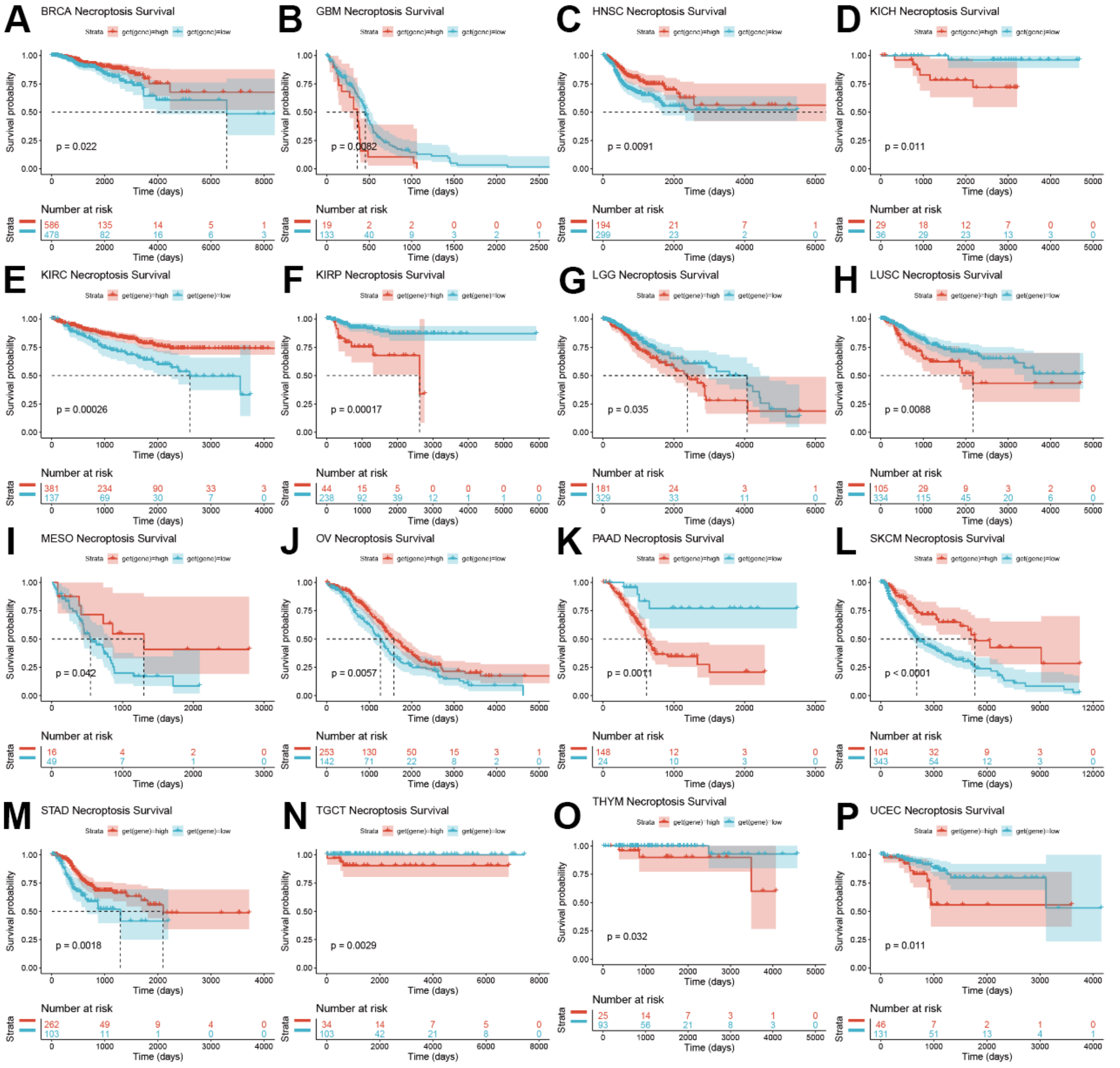

Supplementary Figure 6. Associations between NPS and DSS using Kaplan-Meier method in cancers. (**A**) BRCA. (**B**) GBM. (**C**) HNSC. (**D**) KICH. (**E**) KIRC. (**F**) KIRP. (**G**) LGG. (**H**) LUSC. (**I**) MESO. (**J**) OV. (**K**) PAAD. (**L**) SKCM. (**M**) STAD. (**N**) TGCT. (**O**) THYM. (**P**) UCEC.

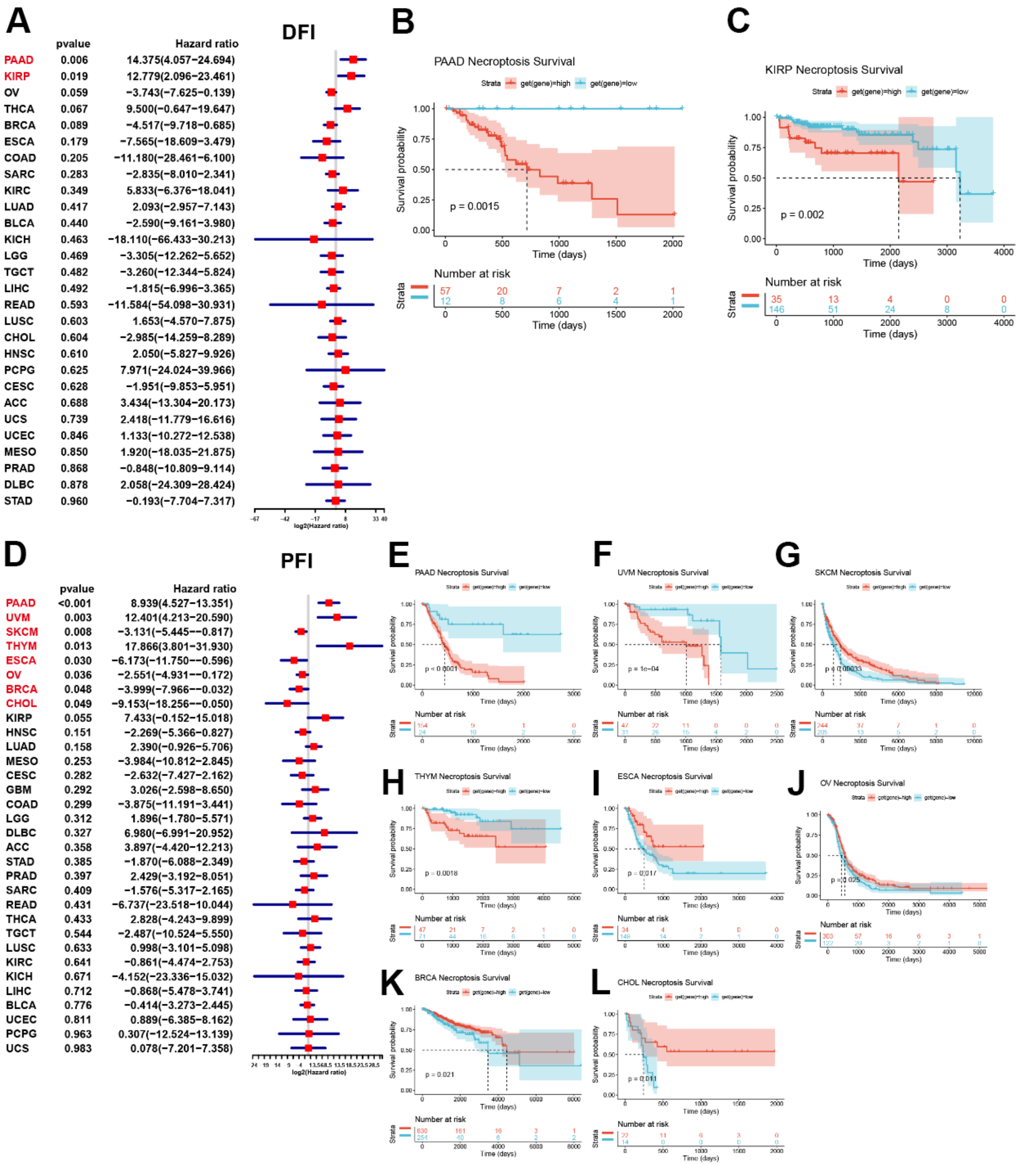

Supplementary Figure 7. Associations between NPS and DFI and PFI in pan-cancer using log-rank method. (**A**) Forest showed the association between NPS and DFI using univariate cox regression. (**B**) PAAD. (**C**) KIRP. (**D**) Forest plot showed the association between NPS and PFI using univariate cox regression. (**E**) PAAD. (**F**) UVM. (**G**) SKCM. (**H**) THYM. (**I**) ESCA. (**J**) OV. (**K**) BRCA. (**L**) CHOL.

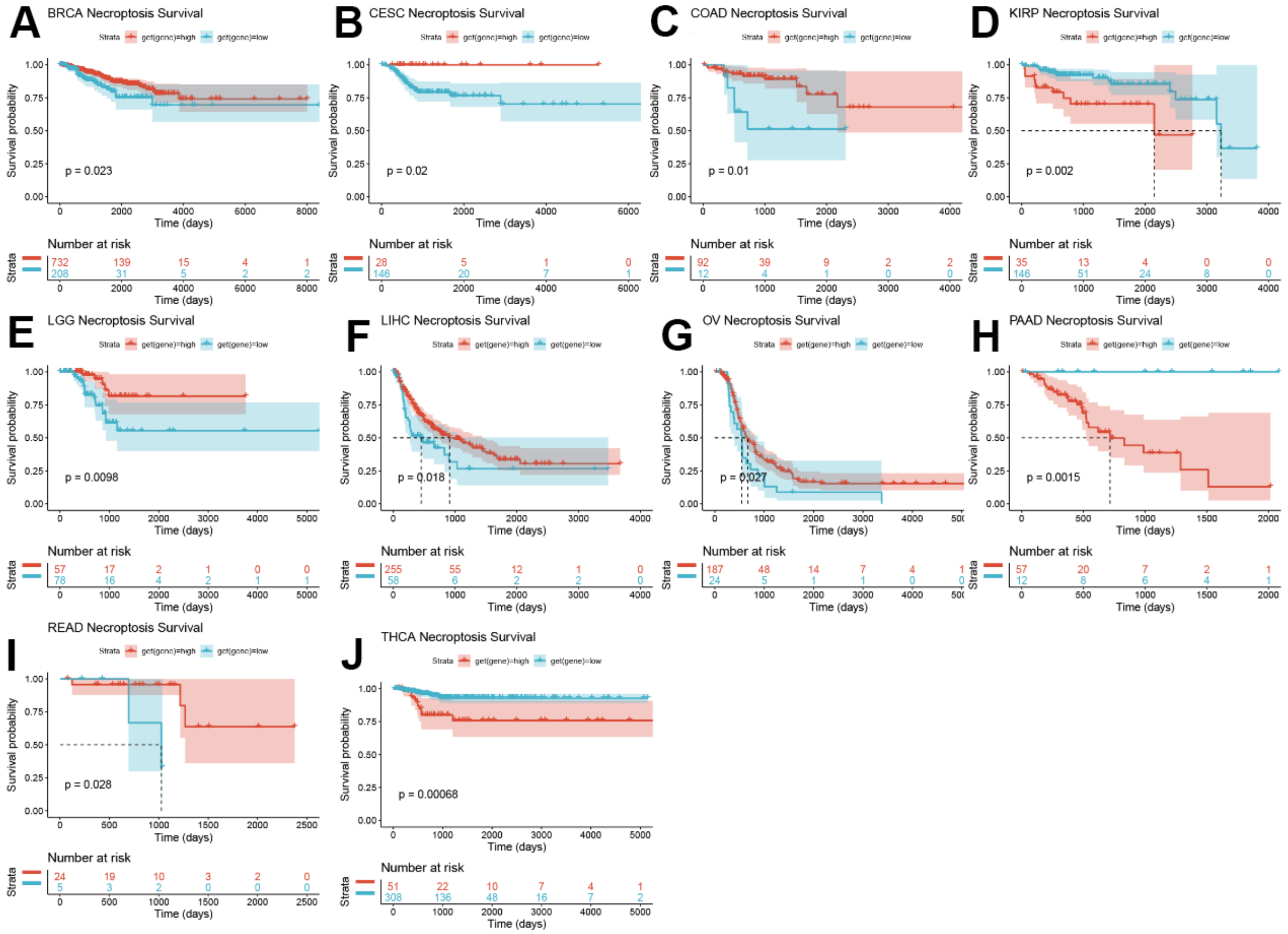

Supplementary Figure 8. Associations between NPS and disease-free interval in pan-cancer using log-rank method. (**A**) BRCA. (**B**) CESC. (**C**) COAD. (**D**) KIRP. (**E**) LGG. (**F**) LIHC. (**G**) OV. (**H**) PAAD. (**I**) READ. (**J**) THCA.

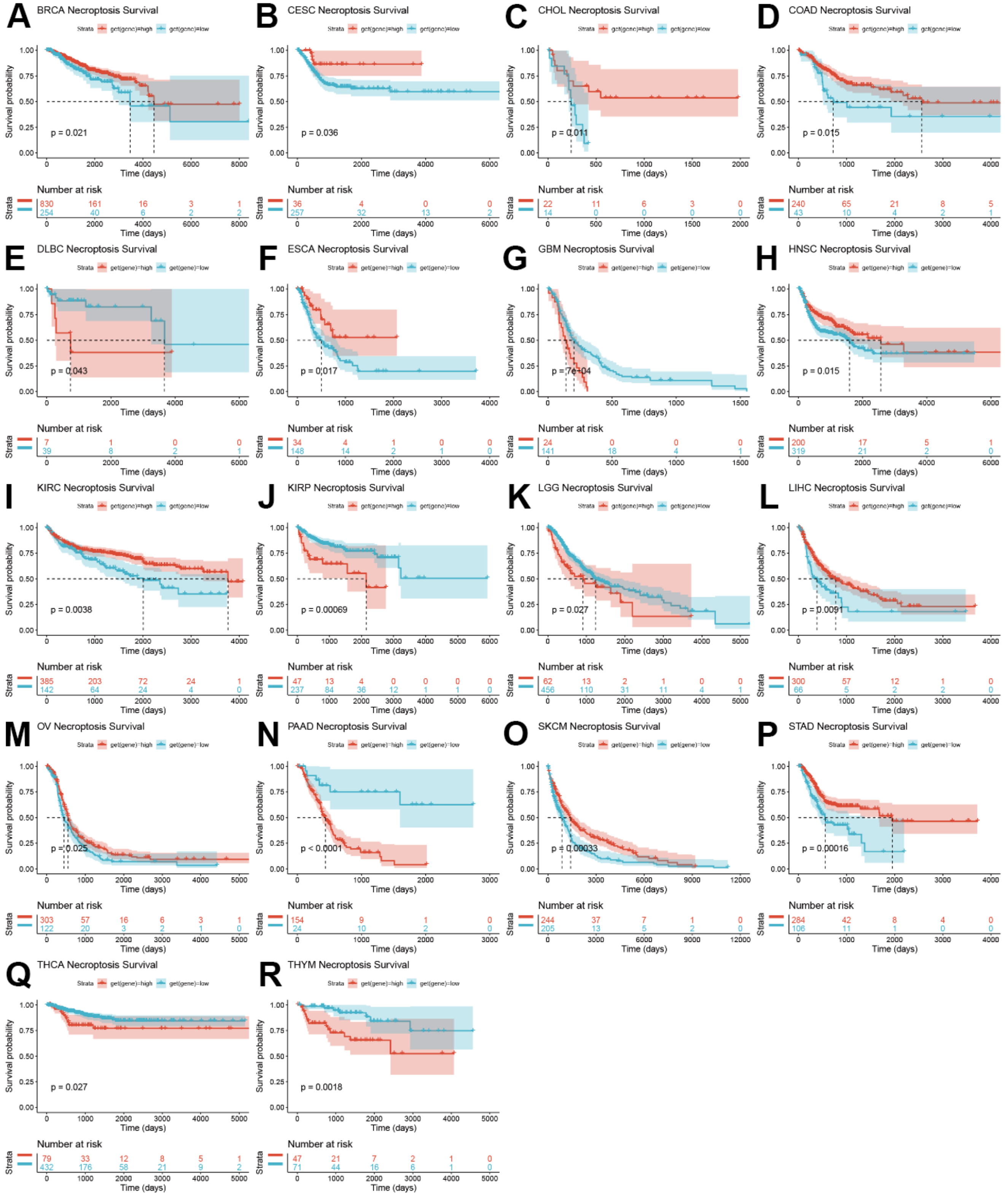

Supplementary Figure 9. Associations between NPS and progression-free survival in pan-cancer using log-rank method. (**A**) BRCA. (**B**) CESC. (**C**) CHOL. (**D**) COAD. (**E**) DLBC. (**F**) ESCA. (**G**) GBM. (**H**) HNSC. (**I**) KIRC. (**J**) KIRP (**K**) LGG. (**L**) LIHC. (**M**) OV. (**N**) PAAD. (**O**) SKCM. (**P**) STAD. (**Q**) THCA. (**R**) THYM.

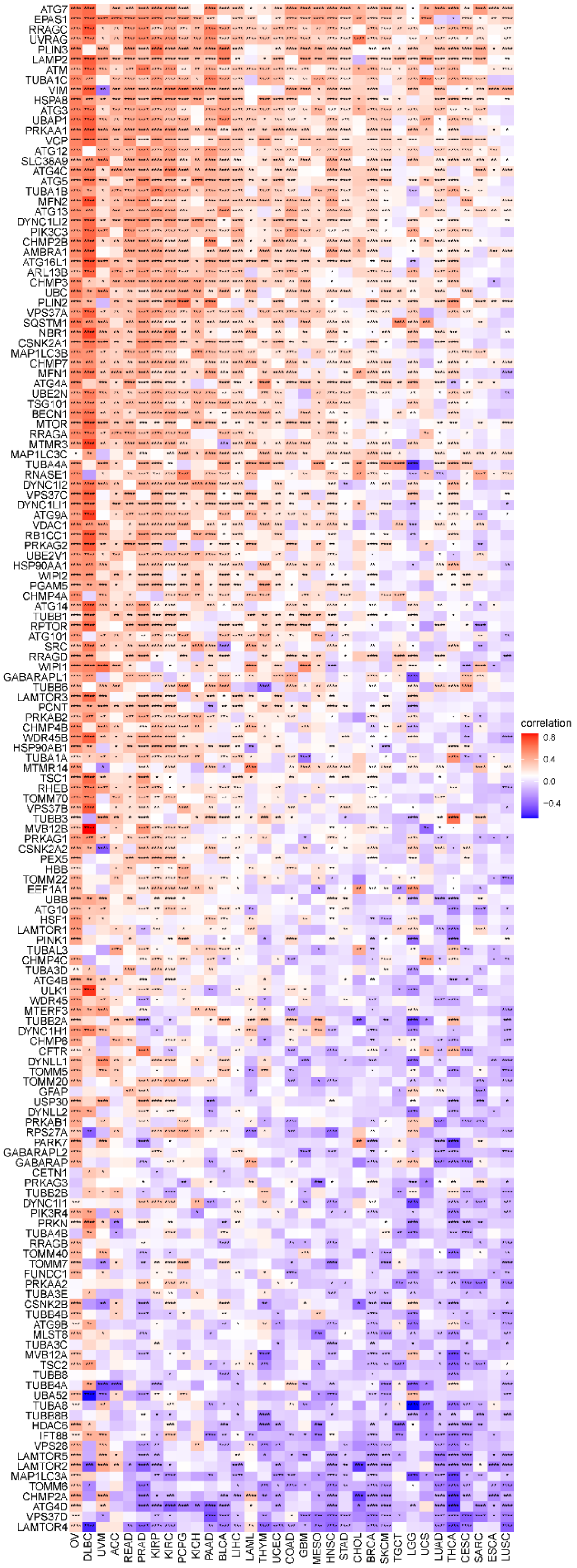

Supplementary Figure 10. Correlations of NPS with autophagy regulators.

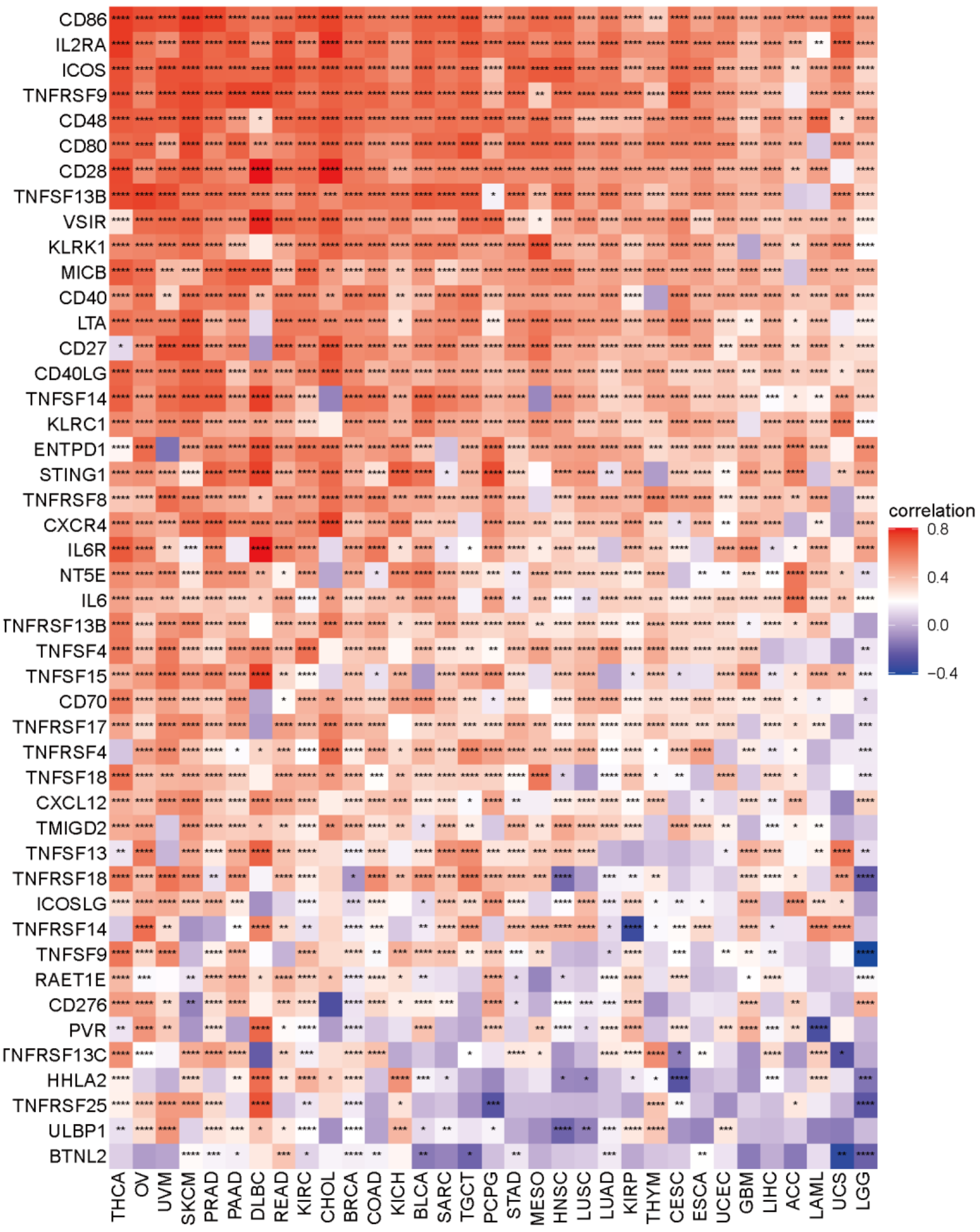

Supplementary Figure 11. Correlations of NPS and immune regulations genes.
