# Supplementary material for: Pan-cancer analysis of a novel indicator of necroptosis with its application in human cancer
Source: Aging (Albany NY). 2022 Sep 26;14(18):7587–616. doi: 10.18632/aging.204307 (PMC9550240; doi:10.18632/aging.204307)
Supplement: Supplementary Tables 1 and 2 [file aging-14-204307-s002.pdf]

SUPPLEMENTARY TABLES

Supplementary Table 1. Lists of cancer types.

Abbreviation	Full name
ACC	Adrenocortical carcinoma
BLCA	Bladder Urothelial Carcinoma
BRCA	Breast invasive carcinoma
CESC	Cervical squamous cell carcinoma and endocervical adenocarcinoma
CHOL	Cholangiocarcinoma
COADREAD	Colon adenocarcinoma/Rectum adenocarcinoma Esophageal carcinoma
DLBC	Lymphoid Neoplasm Diffuse Large B-cell Lymphoma
ESCA	Esophageal carcinoma
GBM	Glioblastoma multiforme
HNSC	Head and Neck squamous cell carcinoma
KICH	Kidney Chromophobe
KIRC	Kidney renal clear cell carcinoma
KIRP	Kidney renal papillary cell carcinoma
LAML	Acute Myeloid Leukemia
LGG	Brain Lower Grade Glioma
LIHC	Liver hepatocellular carcinoma
LUAD	Lung adenocarcinoma
LUSC	Lung squamous cell carcinoma
MESO	Mesothelioma
OV	Ovarian serous cystadenocarcinoma
PAAD	Pancreatic adenocarcinoma
PCPG	Pheochromocytoma and Paraganglioma
PRAD	Prostate adenocarcinoma
READ	Rectum adenocarcinoma
SARC	Sarcoma
SKCM	Skin Cutaneous Melanoma
STAD	Stomach adenocarcinoma
STES	Stomach and Esophageal carcinoma
TGCT	Testicular Germ Cell Tumors
THCA	Thyroid carcinoma
THYM	Thymoma
UCEC	Uterine Corpus Endometrial Carcinoma
UCS	Uterine Carcinosarcoma
UVM	Uveal Melanoma

Supplementary Table 2. The 67 necroptosis regulators used for comprehensive analysis.

Symbol	Entrez gene ID	Description
FADD	8772	Fas Associated Via Death Domain
FAS	355	Fas Cell Surface Death Receptor
FASLG	356	Fas Ligand
MLKL	197259	Mixed Lineage Kinase Domain Like Pseudokinase
RIPK1	8737	Receptor Interacting Serine/Threonine Kinase 1
RIPK3	11035	Receptor Interacting Serine/Threonine Kinase 3
TLR3	7098	Toll Like Receptor 3
TNF	7124	Tumor Necrosis Factor
TSC1	7248	TSC Complex Subunit 1
TRIM11	81559	Tripartite Motif Containing 11
CASP8	841	Caspase 8
ZBP1	81030	Z-DNA Binding Protein 1
MAPK8	5599	Mitogen-Activated Protein Kinase 8
IPMK	253430	Inositol Polyphosphate Multikinase
ITPK1	3705	Inositol-Tetrakisphosphate 1-Kinase
SIRT3	23410	Sirtuin 3
MYC	4609	MYC Proto-Oncogene
TNFRSF1A	7132	TNF Receptor Superfamily Member 1A
TNFSF10	8743	TNF Superfamily Member 10
TNFRSF1B	7133	TNF Receptor Superfamily Member 1B
TRAF2	7186	TNF Receptor Associated Factor 2
PANX1	24145	Pannexin 1
OTULIN	90268	OTU Deubiquitinase With Linear Linkage Specificity
CYLD	1540	CYLD Lysine 63 Deubiquitinase
USP22	23326	Ubiquitin Specific Peptidase 22
MAP3K7	6885	Mitogen-Activated Protein Kinase Kinase Kinase 7
SQSTM1	8878	Sequestosome 1
STAT3	6774	Signal Transducer And Activator Of Transcription 3
DIABLO	56616	Diablo IAP-Binding Mitochondrial Protein
DNMT1	1786	DNA Methyltransferase 1
CFLAR	8837	ASP8 And FADD Like Apoptosis Regulator
BRAF	673	B-Raf Proto-Oncogene, Serine/Threonine Kinase
AXL	558	AXL Receptor Tyrosine Kinase
ID1	3397	Inhibitor Of DNA Binding 1
CDKN2A	1029	Cyclin Dependent Kinase Inhibitor 2A
HSPA4	3308	Heat Shock Protein Family A (Hsp70) Member 4
BCL2	596	BCL2 Apoptosis Regulator
STUB1	10273	STIP1 Homology And U-Box Containing Protein 1
FLT3	2322	Fms Related Receptor Tyrosine Kinase 3
HAT1	8520	Histone Acetyltransferase 1
SIRT2	22933	Sirtuin 2
SIRT1	23411	Sirtuin 1
PLK1	5347	Polo Like Kinase 1
MPG	4350	N-Methylpurine DNA Glycosylase
BACH2	60468	BTB Domain And CNC Homolog 2
GATA3	2625	GATA Binding Protein 3
MYCN	4613	MYCN Proto-Oncogene, BHLH Transcription Factor
ALK	238	ALK Receptor Tyrosine Kinase

ATRX	546	ATRX Chromatin Remodeler
TERT	7015	Telomerase Reverse Transcriptase
SLC39A7	7922	Solute Carrier Family 39 Member 7
SPATA2	9825	Spermatogenesis Associated 2
RNF31	55072	Ring Finger Protein 31
IDH1	3417	Isocitrate Dehydrogenase (NADP(+)) 1
IDH2	3418	Isocitrate Dehydrogenase (NADP(+)) 2
KLF9	687	Kruppel Like Factor 9
HDAC9	9734	Histone Deacetylase 9
HSP90AA1	3320	Heat Shock Protein 90 Alpha Family Class A Member 1
LEF1	51176	Lymphoid Enhancer Binding Factor 1
BNIP3	664	BCL2 Interacting Protein 3
CD40	958	CD40 Molecule
BCL2L11	10018	BCL2 Like 11
EGFR	1956	Epidermal Growth Factor Receptor
DDX58	23586	DExD/H-Box Helicase 58
TARDBP	23435	TAR DNA Binding Protein
APP	351	Amyloid Beta Precursor Protein
TNFRSF21	27242	TNF Receptor Superfamily Member 21
